# Cell surface–tethered IL-12 repolarizes the tumor immune microenvironment to enhance the efficacy of adoptive T cell therapy

**DOI:** 10.1126/sciadv.abi8075

**Published:** 2022-04-27

**Authors:** Douglas S. Jones, Jonathan D. Nardozzi, Katharine L. Sackton, Gulzar Ahmad, Esben Christensen, Lars Ringgaard, De-Kuan Chang, Ditte E. Jaehger, Jacob V. Konakondla, Martin Wiinberg, Kate L. Stokes, Alvin Pratama, Karsten Sauer, Thomas L. Andresen

**Affiliations:** 1 Repertoire Immune Medicines, Cambridge MA, USA.; 2 Technical University of Denmark, Copenhagen, Denmark.

## Abstract

Immune-activating cytokines such as interleukin-12 (IL-12) hold strong potential for cancer immunotherapy but have been limited by high systemic toxicities. We describe here an approach to safely harness cytokine biology for adoptive cell therapy through uniform and dose-controlled tethering onto the surface of the adoptively transferred cells. Tumor-specific T cells tethered with IL-12 showed superior antitumor efficacy across multiple cell therapy models compared to conventional systemic IL-12 coadministration. Mechanistically, the IL-12–tethered T cells supported a strong safety profile by driving interferon-γ production and adoptively transferred T cell activity preferentially in the tumor. Immune profiling revealed that the tethered IL-12 reshaped the suppressive tumor immune microenvironment, including triggering a pronounced repolarization of monocytic myeloid-derived suppressor cells into activated, inflammatory effector cells that further supported antitumor activity. This tethering approach thus holds strong promise for harnessing and directing potent immunomodulatory cytokines for cell therapies while limiting systemic toxicities.

## Teaser

Controlled surface tethering of IL-12 enhances cell therapy by boosting T cell function and repolarizing tumor immune activity.

## INTRODUCTION

Adoptive T cell therapies (ACTs) have proven to be life-saving medicines for certain types of cancers (*1*). Clinical results have been most prominent for chimeric antigen receptor T cell (CAR-T) therapy targeting CD19 on leukemia and lymphoma cells, which has resulted in a high proportion of complete durable remissions (*2*, *3*). Other ACT approaches, such as tumor-infiltrating lymphocyte (TIL) or T cell receptor–engineered T cell (TCR-T) therapies, have delivered clinical responses in melanoma (*4*, *5*), human papilloma virus–associated epithelial cancers (*6*–*9*), and sarcoma (*5*). However, these responses have been short-lived, and delivering durable responses in solid tumors constitutes a major clinical challenge (*10*).

The immunosuppressive tumor microenvironment (TME) is believed to be a key factor limiting the efficacy of T cell therapies for solid tumors (*10*). When solid tumors have progressed to be clinically detectable, they have typically evaded the antitumor immune response by suppressing either immune recognition or T cell activity (*11*). Preclinical and clinical studies suggest roles for inhibitory cells in the tumor, such as regulatory T cells, myeloid-derived suppressor cells (MDSCs), or tumor-associated macrophages (TAMs) (*11*–*14*), and up-regulation of inhibitory costimulatory molecules (*15*–*19*) as key suppressive mechanisms mediating immune evasion. Immune-activating agents that enhance T cell activity or repolarize the immunosuppressive microenvironment hold promise for broadening the reach of T cell therapies and are thus an intense area of research.

Cytokines such as interleukin-2 (IL-2), IL-12, and IL-15 have been explored for improving antitumor activity of ACT (*10*). IL-12 is particularly attractive for such a purpose given its potent immune-activating properties (*20*, *21*). It is a heterodimeric protein, composed of p35 (IL-12A) and p40 (IL-12B) subunits, that was originally characterized as a potent activator of natural killer (NK) cells (*22*). IL-12 has since been shown to also promote differentiation of CD4 T cells to interferon-γ (IFN-γ)–producing type 1 helper cells (T_H_1), increase CD8 T cell cytotoxicity, up-regulate antigen presentation, and reprogram MDSCs to a T cell–supportive phenotype (*23*–*26*).

The clinical utility of IL-12, however, has been limited by severe toxicities upon systemic administration (*27*–*29*). In an effort to safely harness IL-12 for cancer therapy, several groups have investigated the ability to stimulate antitumor immune responses selectively in the TME. This includes efforts to genetically engineer tumor-specific T cells to drive IL-12 expression selectively upon tumor antigen encounter (*30*). This markedly improved the efficacy of T cell therapy in a mouse tumor model (*25*, *30*). Clinical evaluation of TILs gene-engineered to produce IL-12 in this manner resulted in objective clinical responses at 10- to 100-fold lower cell doses than those required for historical TIL therapy, including in a patient that previously failed the standard TIL therapy (*28*). However, despite the encouraging efficacy, insufficient control of IL-12 expression across patients resulted in severe IFN-γ–related toxicities and further development of this approach was halted (*28*). Gene-engineered production of IL-12 also substantially complicated TIL manufacturing since sustained exposure to IL-12 drives T cell dysfunction (*28*). This work nevertheless demonstrates the therapeutic potential of IL-12 in conjunction with T cell therapy. Taken together, safely harnessing IL-12 for cancer immunotherapy requires strong pharmacologic control of IL-12 dosing and its activity must also be focused toward the TME (*31*).

Here, we describe a novel approach to control cytokine dose-level and activity profile by directly tethering the cytokine to the surface of tumor-specific T cells before adoptive transfer. This is achieved by directly tethering to highly expressed cell surface receptors, which provides improved regulation and control over potent immune-activating cytokines. Since the surface tethering is performed after cell manufacture, this approach is compatible with TIL, CAR-T, and TCR-T therapies. We demonstrate the therapeutic potential of this approach using tethered IL-12 in multiple cell therapy models and report improved antigen-specific T cell function and antitumor efficacy accompanied by modulation of the tumor-immune microenvironment with minimal systemic effects.

## RESULTS

### Controlled and uniform tethering of cytokines to the T cell surface

We reasoned that systemic cytokine toxicities can be mitigated by directly tethering cytokines to the immune surface receptors before adoptive transfer. To tether a functionally relevant dose of cytokine to T cells, surface receptors in greater abundance than cytokine signaling receptors are required. While receptors for potent immune-activating cytokines are generally present at low levels on the T cell surface, integrins and adhesion receptors involved in transient cell-cell interactions, for example, are typically present in high abundance. We therefore profiled the expression of a range of cell surface molecules on T cells from three human donors by quantifying binding of fluorescent-labeled antibodies (fig. S1).

**Fig. 1.**
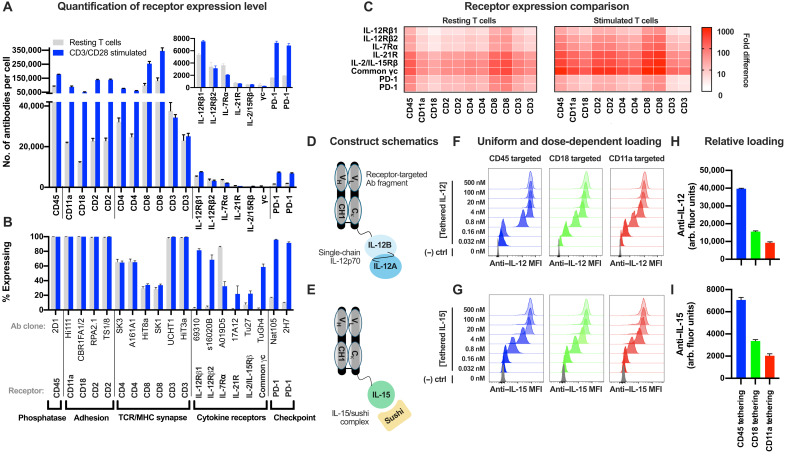
Cytokine tethering to abundant cell surface receptors. (**A** and **B**) Quantification of the number of receptor-targeted antibodies bound per cell (A) or percent of cells expressing the given receptor (B) on resting (gray) or CD3/CD28-stimulated (blue) T cells. In (A), only the expressing population of cells is included for analysis; for example, CD8-negative cells are excluded from analysis of CD8 antibody binding capacity in (A). Inset: Antibodies per cell for cytokine and checkpoint receptors with enlarged *y* axis. Error bars represent SD for T cells from three human donors. Multiple antibody clones targeting the same receptor were used when available to improve accuracy. (**C**) Fold difference of the average number of antibodies bound per cell for the abundant receptors versus cytokine and checkpoint receptors. Antibody clones are in the same order as in (A) and (B). (**D** to **I**) Construct design (D and E), dose-dependent loading (F and G), and relative cytokine loading at 500 nM condition (H and I) for surface-tethered IL-12 and IL-15 constructs. Variable domains for anti-CD11a, anti-CD18, and anti-CD45 antibodies were from antibody clones MHM24, 1B4, and 9.4, respectively. PD-1, programmed cell death protein 1; MHC, major histocompatibility complex; MFI, median fluorescent intensity; Ab, antibody.

Antibody binding capacity for cytokine receptors ranged from 250 to 8000 antibodies per T cell, while binding capacity for the integrin molecules CD2, CD11a, and CD18 ranged from 12,000 to 150,000 antibodies per T cell (Fig. 1, A and B). The expression of these integrin molecules and of co-receptors (CD4 and CD8) was higher on T cells activated by CD3/CD28 stimulation than on resting T cells (Fig. 1, A and B). With the exception of IL-7Rα, cytokine receptors were expressed on an increased proportion of the activated T cells (Fig. 1B), but the number of receptors per cell within the expressing population of T cells was similar between resting and activated T cells (Fig. 1A, inset). As expected, the proportion of cells expressing programmed cell death protein 1 (PD-1) was also increased following activation, but its overall expression level remained low and was similar to that of the cytokine receptors. The cytokine receptors were present at 10- to 1000-fold lower levels than adhesion molecules, TCR complex and co-receptors, and CD45 phosphatase, with consistent results across three human donors and between resting T cells and T cells activated with CD3/CD28 stimulation (Fig. 1C). Together, this demonstrates that multiple receptors are expressed at orders of magnitude higher levels than cytokine receptors and that they may serve as “handles” for cell surface cytokine tethering.

To examine whether these highly expressed receptors characterized above could be used to tether cytokines to the cell surface, we constructed cytokine “tethered fusion proteins” by linking a cytokine to a receptor-targeted antibody. Tethered fusions composed of IL-12 or IL-15 and antibodies specific for CD11a, CD18, or CD45 were constructed by genetically fusing the cytokine to the C terminus of the respective antigen binding fragment (Fab) antibody light chain using a flexible polypeptide linker (Fig. 1, D and E). For IL-12–tethered fusions, genetically fusing the p35 and p40 subunits enabled the expression of IL-12 from a single open reading frame (*32*). For IL-15–tethered fusions, the cytokine was noncovalently associated with the sushi domain of the IL-15 receptor α subunit, which enhances IL-15 function (*33*, *34*). Each cell surface receptor facilitated uniform, dose-dependent loading of the cytokines onto the T cell surface (Fig. 1, F and G). For both cytokines, CD45 yielded higher tethering than CD11a or CD18 (Fig. 1, H and I). As controls, incubating a CD45-tethered IL-12 with Jurkat T cells subjected to CD45 knockdown resulted in minimal tethered fusion loading compared to parental cells, and native IL-12 alone resulted in negligible binding compared to CD45-tethered IL-12 (fig. S2). Together, this demonstrates that antibody-mediated tethering to abundant cell surface receptors can load doses of cytokines onto the T cell surface that exceed the number of the respective cytokine receptors on the given cell.

### Cellular tethering provides a reservoir of bioactive cytokine

**Fig. 2.**
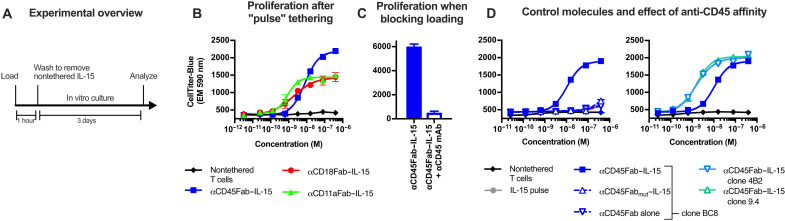
Tethering to abundant surface receptors provides a cell surface cytokine reservoir. (**A**) Schematic for pulse bioassay. (**B**) T cell proliferation 3 days after pulse incubation with serial dilutions of CD45-, CD18-, or CD11a-targeted IL-15/sushi fusions. Variable domains for anti-CD11a, anti-CD18, and anti-CD45 antibodies are from antibody clones MHM24, 1B4, and BC8, respectively. (**C**) T cell proliferation induced by the CD45-tethered IL-15/sushi fusion after pulse incubation in the presence or absence of competing anti-CD45 antibody. (**D**) Comparison of T cell proliferation after pulse incubation with nontethered IL-15/sushi complex, anti-CD45 Fab antibody alone, or fusion of IL-15/sushi complex to mutated/nonbinding anti-CD45 Fab antibody (left) or from fusion of IL-15/sushi complex to antibodies with increased CD45 affinity (right, clones 4B2 and 9.4). T cells alone and fusion to BC8 antibody clone are replotted on left and right plots of (D) for reference. All data are representative of experiments performed on at least two separate days.

To determine whether such cytokine loading could provide a sustained stimulus to the tethered cells, we evaluated T cell expansion following pulse incubation with IL-15–tethered fusions targeting CD11a, CD18, or CD45, in which unbound tethered fusion protein was removed by washing before plating the cells in culture. Each of the tethered fusions facilitated dose-dependent T cell expansion (Fig. 2, A and B). As a control, blocking tethered fusion loading by coincubation with a molar excess of free anti-CD45 antibody completely ablated the proliferative effect (Fig. 2C). As further controls, pulse incubation with free IL-15, anti-CD45 antibody alone (without IL-15 fusion), or a CD45-tethered IL-15 containing a mutated, nonbinding antibody region did not induce T cell expansion above background (Fig. 2D, left). IL-15–tethered fusions containing higher-affinity antibodies had stronger potency in this assay, demonstrating the importance of antibody affinity in cellular tethering (Fig. 2D, right). In addition, pulse incubation with CD45-tethered IL-15, but not free IL-15, resulted in similar activation of signal transducers and activators of transcription 5 (STAT5), a transcription factor downstream of IL-15 receptors, as constant incubation with free IL-15 (fig. S3, A and C). Similar results were observed for activation of STAT4, a transcription factor downstream of IL-12 receptors, following pulse incubation with CD45-tethered IL-12 (fig. S3, B and D). Treatment of nonloaded T cells with conditioned supernatant from cells tethered with IL-12 also resulted in STAT4 activation. This indicates that the tethered cytokines can dissociate from the loaded T cell and activate other T cells in a paracrine manner. We conclude that the tethered fusions provide a modular approach to load a reservoir of bioactive cytokine on the T cell surface, which can then act in an autocrine or paracrine manner.

### Cell-tethered IL-12 enhances antigen-specific T cell activity and cytotoxicity in vitro

**Fig. 3.**
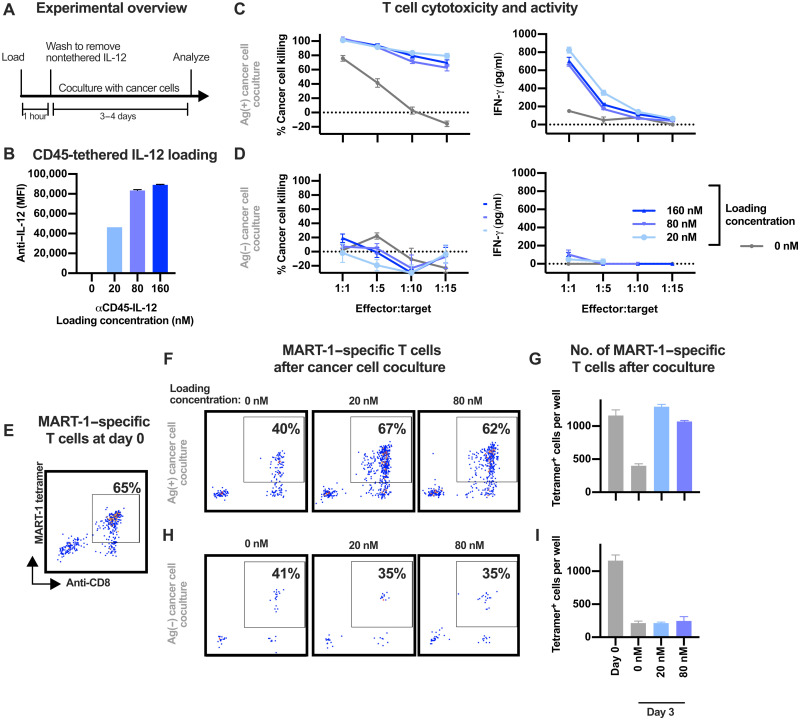
IL-12 tethering enhances tumor-specific T cell activity and function specifically in presence of cognate antigens. (**A**) Schematic for coculture assay. (**B**) Relative loading onto MART-1–specific T cells from various concentrations of CD45-tethered IL-12 assessed by flow cytometry. Antibody variable domains are from a humanized form of clone 9.4. (**C** and **D**) Cancer cell killing (left) or IFN-γ production (right) by MART-1–specific T cells following 4 days of coincubation at various T cell effector to target cancer cell ratios with melanoma cells that express [SK-MEL-5 cells (C)] or do not express [A375 cells (D)] the MART-1 antigen. (**E**, **F**, and **H**) Proportion of T cells specific for the MART-1 antigen before (E) or after 3 days of coincubation with SK-MEL-5 (F) or A375 (H) cancer cells (effector:target ratio of 1:5). Axis labels in all panels are the same as in (E). (**G** and **I**) Number of MART-1–specific T cells in culture following coincubation with MART-1–expressing SK-MEL-5 cells (G) or non–MART-1–expressing A375 cells (I). All data are representative of experiments performed on at least three separate days.

Given the potent immune-activating potential of IL-12, we evaluated whether T cell–tethered IL-12 could enhance T cell activity without driving off-target activity. We used CD45-tethering based on its high relative loading capacity compared to other cell surface receptors, as characterized above. Human CD8 T cells specific for the immunodominant HLA (human leukocyte antigen)–A*02:01–restricted melanoma antigen recognized by T-cells (MART-1) peptide ELAGIGILTV (*35*) were first generated by ex vivo stimulation of T cells from an HLA-A*02:01 human donor with autologous monocytic-derived dendritic cells (mDCs) presenting the MART-1 peptide. In coculture with the HLA-A*02:01–positive MART-1–expressing human melanoma cell line SK-MEL-5, the MART-1–specific CD8 T cells loaded with CD45-tethered IL-12 showed increased cytolysis of the target cancer cells without an apparent dose dependence for the levels of IL-12 loaded on the T cells (Fig. 3, A to C). IFN-γ production, a key effector response of antigen-activated T cells, was also increased compared to MART-1–specific T cells alone (Fig. 3C). Coincubation with A375 cells, an HLA-A*02:01–positive human melanoma cell line that does not express the MART-1 antigen (*36*), did not elicit increased cytokine production or cytolytic activity from the T cells (Fig. 3D), demonstrating that the enhanced T cell activity from IL-12 surface tethering is antigen specific. To evaluate the effects of tethered IL-12 on T cell viability, the presence of antigen-specific CD8 T cells following coincubation with the cancer cell lines was assessed by staining with MART-1 major histocompatibility complex (MHC) tetramers. Compared to nontethered T cells, the CD45-tethered IL-12 maintained the viability of the MART-1–specific T cells when coincubated with the MART-1–expressing SK-MEL-5 cells (Fig. 3, E to G) but not when coincubated with the control A375 cells that do not express the MART-1 antigen (Fig. 3, H and I). Together, these data demonstrate that cell tethered IL-12 improves the function, cytotoxicity, and survival of tumor-specific human T cells in an antigen-specific manner.

### Cell-tethered IL-12 safely enhances the efficacy of tumor-specific adoptive T cell therapy

**Fig. 4.**
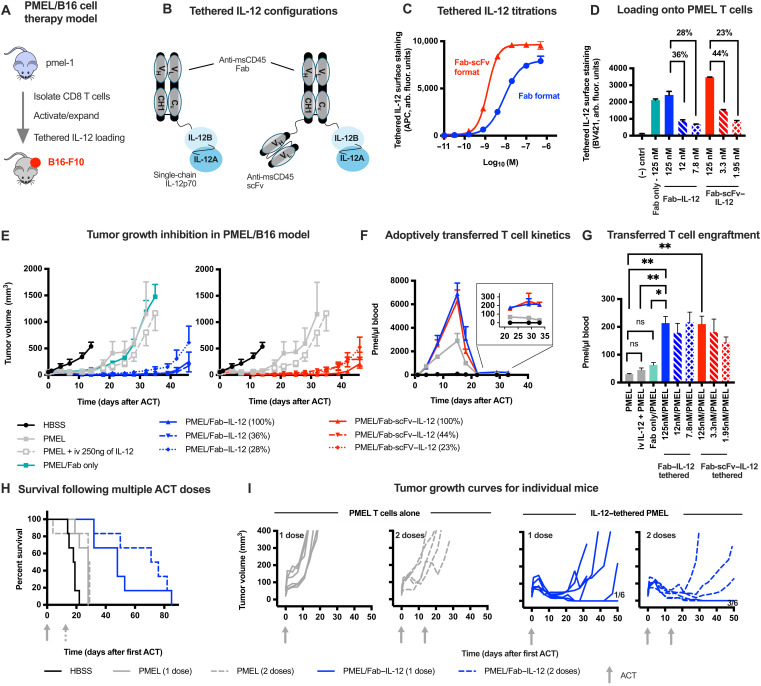
IL-12 tethering enhances adoptive cell therapy antitumor efficacy. (**A**) Schematic for PMEL/B16 T cell therapy model. CD8 T cells are isolated from *pmel-*1 mice, activated and expanded ex vivo, and then transferred into B16-F10 tumor-bearing C57BL6/J mice with or without IL-12 tethering before adoptive transfer. Mice were preconditioned with cyclophosphamide 1 day before adoptive transfer. (**B**) Schematic of CD45-tethered IL-12 constructs comprising single-chain mouse IL-12p70 fused to an anti-mouse CD45Fab (left, Fab–IL-12) or anti-mouse CD45Fab-scFv (right, Fab-scFv–IL-12). Antibody variable domains are from clone M1/9.3.4.HL.2. (**C**) Titration of Fab–IL-12 and Fab-scFv–IL-12 onto mouse T cells, assessed by flow cytometry. (**D**) Relative loading of anti-CD45Fab antibody alone or of CD45-tethered IL-12 constructs onto PMEL T cells, assessed by flow cytometry before adoptive transfer. (**E**) Tumor growth curves following treatment of mice bearing intradermal B16-F10 tumors with 5 × 10^6^ PMEL T cells containing varied amounts of tethered IL-12. PMEL T cells and cell-free IL-12 comparators were each administered on day 0. Data are plotted until two mice in a given group reach euthanasia criteria. (**F**) Circulating PMEL T cells following adoptive transfer. Inset: Enlarged *y* axis comparing longer-term engraftment. (**G**) Circulating PMEL T cells across all study groups 33 days after adoptive transfer. (**H** and **I**) Antitumor efficacy from one or two doses of 5 × 10^6^ PMEL T cells alone or tethered with IL-12 evaluated by overall survival (H) or individual tumor growth curves (I). Values on the right plots of (I) indicate proportion of mice with smaller tumors at day 50 than their starting tumor volume; gray arrows indicate dosing time points. Cyclophosphamide preconditioning preceded only the first PMEL T cell dose, and the second dose was administered without further preconditioning. T cell engraftment data were compared by one-way analysis of variance (ANOVA) with a Tukey’s posttest. ns, not significant. HBSS, Hanks’ balanced salt solution. *P < 0.05 and **P < 0.01.

We next evaluated whether cell-tethered IL-12 could enhance the activity of adoptively transferred tumor-specific T cells in vivo. We used an immune-competent mouse model that allowed us to evaluate both potential IL-12–driven toxicities and the IL-12 activity on endogenous immune cells in the tumor. Briefly, CD8 T cells from pmel-1 TCR transgenic mice (PMEL T cells), which are specific for the gp100 antigen expressed by B16-F10 mouse melanoma cells (*37*), were activated and expanded ex vivo and then tethered with IL-12 before adoptive transfer into mice bearing B16-F10 tumors (Fig. 4A). This model required the generation of a mouse IL-12–tethered fusion since human IL-12 does not activate mouse IL-12 receptors, and the anti-human CD45 Fab antibody does not cross-react with mouse CD45 (fig. S4, A and B). The CD45-tethered IL-12 loaded approximately 42 ng of IL-12 per 10^6^ mouse T cells, which is comparable to human CD45-tethered IL-12 loading onto human T cells (fig. S4, C and D). Adoptive transfer of 5 × 10^6^ PMEL T cells loaded with CD45-tethered IL-12 exhibited enhanced antitumor efficacy compared to PMEL T cells alone, both when delivered systemically by intravenous administration or when delivered directly to the tumor by intratumoral administration (fig. S5). Efficacy of the IL-12–tethered T cells was similar upon both systemic and intratumoral administration.

We next generated two forms of CD45-tethered IL-12 to evaluate the effects of tethered fusions with different CD45 binding affinities. One form consisted of a monovalent anti-mouse CD45 Fab antibody fragment and the other used bivalent Fab and scFv (single-chain variable fragment) fusion format (Fab-scFv) that had approximately sixfold higher CD45 binding affinity than the Fab format due to increased binding valency (Fig. 4, B and C). Each form of the CD45-tethered IL-12 enhanced antitumor efficacy compared to adoptive transfer of 5 × 10^6^ PMEL T cells alone, including PMEL T cells tethered with subsaturating levels of the CD45-tethered IL-12 constructs (Fig. 4, D and E). In the case of the CD45Fab-tethered IL-12, the three doses examined correspond to approximately 60, 80, and 210 ng of IL-12, for 28, 36, and 100% of saturated loading, respectively, based on tethered IL-12 T cell loading (fig. S4C). By comparison, PMEL T cells coadministered with a fourfold higher dose of direct intravenously injected IL-12 did not meaningfully enhance tumor growth inhibition in this model (Fig. 4E). This demonstrates that tethered IL-12 enhances T cell antitumor efficacy more effectively than coadministered systemic IL-12. Similar tumor growth inhibition by the IL-12–tethered fusions containing antibody regions with varied affinity (monovalent Fab or the bivalent Fab-scFv binding regions; Fig. 4E) suggests that the Fab antibody fragment used here has sufficient affinity to provide the improved antitumor activity. Analysis of tumor growth curves from individual animals demonstrated that while subsaturated loading of IL-12 onto the tumor-specific T cells enhanced tumor growth inhibition, higher IL-12 loading trended toward deeper and more durable tumor regression for both the Fab- and Fab-scFv–tethered IL-12 formats (fig. S6). As a control, tethering the anti-CD45 Fab antibody fragment alone did not affect PMEL T cell antitumor activity (Fig. 4E). This shows that cell-tethered IL-12—rather than the anti-CD45 antibody fragment—is critical for delivering the observed efficacy and that antibody tethering to CD45 does not impair T cell function.

At the cellular level, the CD45-tethered IL-12 resulted in increased peak expansion of the adoptively transferred T cells compared to transfer of T cells alone, and similar kinetics were observed for both the Fab and Fab-scFv formats of the CD45-tethered IL-12 (Fig. 4F). Each tethered IL-12 construct also increased long-term engraftment about 7.0-fold compared to adoptive transfer of T cells alone (213 ± 24 cells/μl for Fab and 210 ± 28 cells/μl for Fab-scFv formats compared to 30 ± 2 cells/μl for T cells alone) as measured from circulating PMEL T cell levels 33 days after adoptive transfer (Fig. 4G). The tethered IL-12 increased engraftment 4.7-fold compared to coadministration with direct intravenously injected IL-12 (45 ± 7 cells/μl). These effects were observed without apparent dose dependence for surface-tethering levels, as subsaturating IL-12 loading resulted in similar long-term engraftment as saturating IL-12 loading (Fig. 4G). Tethering the anti-CD45 Fab antibody fragment alone did not significantly improve engraftment. Together, this demonstrates that cell-tethered IL-12 supports engraftment of the adoptively transferred cells more effectively than an at least fourfold higher dose of native IL-12.

Improved efficacy and T cell engraftment were observed in the absence of overt toxicities such as body weight loss (fig. S7A). Consistent with this, induction of systemic IFN-γ was also moderate and transient, peaking at 200 to 300 pg/ml 1 day after adoptive transfer and returning to baseline within 4 to 8 days of ACT (fig. S7B). Escalating the cell dose to 40 × 10^6^ IL-12–tethered T cells resulted in a transient, peak mouse body weight loss of 3.4 ± 1.9% 4 days after adoptive transfer that reversed to baseline by day 7 (fig. S8A). Liver and kidney toxicity have been key dose-limiting toxicities of IL-12 in clinical studies (*28*, *38*, *39*). None of the doses examined resulted in changes in blood urea nitrogen or aspartate aminotransferase, two circulating biomarkers of kidney and liver toxicity, respectively (fig. S8B). Alanine aminotransferase, another biomarker of liver toxicity, showed a moderate increase up to twofold 4 days after ACT compared to vehicle control, which returned to baseline levels by day 8 (fig. S8B). This favorable safety profile allowed us to further improve antitumor efficacy by administering multiple doses of IL-12–tethered T cells (Fig. 4, H and I). The improved antitumor efficacy was characterized by increased durability of tumor regression compared to a single dose of IL-12–tethered tumor-specific T cells (Fig. 4, H and I). By comparison, multiple doses of tumor-specific T cells alone did not improve antitumor efficacy compared to a single dose. Together, this demonstrates that cell-tethered IL-12 safely enhances the efficacy of T cell therapy in an immune competent mouse model.

### Cell-tethered IL-12 improves potency in a multitargeted T cell therapy model

**Fig. 5.**
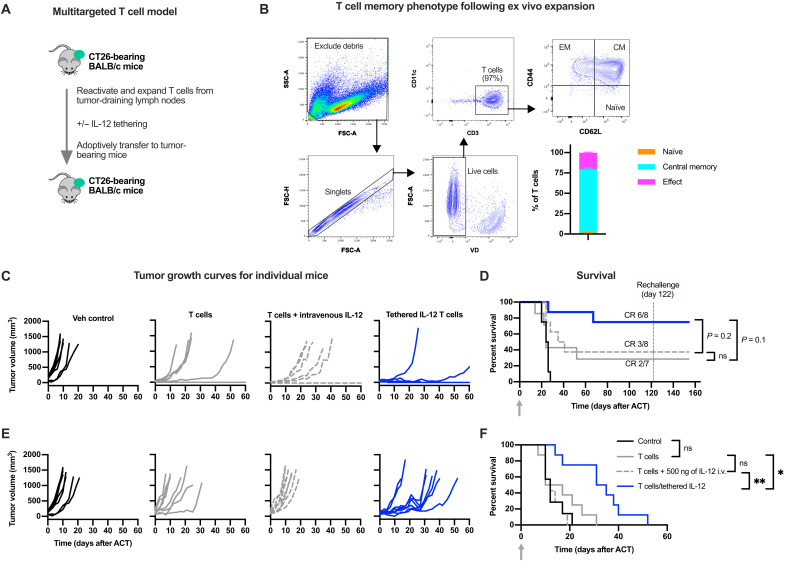
Enhanced antitumor efficacy in an endogenous, multitargeted T cell therapy model. (**A**) Schematic of the model. T cells were isolated from tdLNs of CT26 tumor-bearing BALB/c mice, reactivated, expanded and tethered to IL-12 or not ex vivo, and then adoptively transferred into BALB/c mice bearing subcutaneous CT26 tumors. This model was run in the absence of lymphodepleting preconditioning. (**B**) Multicolor flow cytometric gating strategy to assess T cell memory phenotype following reactivation and expansion. CM, central memory; EM, effective memory. (**C** to **F**) Antitumor efficacy of a single dose of 5 × 10^6^ T cells administered into mice bearing smaller [30-mm^3^ average starting tumor volume (C and D)] or larger [80-mm^3^ average starting tumor volume (E and F)] CT26 tumors. Nontethered mouse IL-12 was dosed intravenously (500 ng); CD45-tethered IL-12 corresponds to approximately 210 ng of IL-12 based on the average T cell loading. Survival was compared by a log-rank Mantel-Cox test. *P < 0.05 and **P < 0.01. FSC, forward scatter; SSC, side scatter; VD, viability dye; CR, complete response.

Antigen heterogeneity in solid tumors is a major obstacle for single antigen-targeted T cell therapies (*40*, *41*), and T cell therapies that recognize multiple tumor antigens are a major emphasis of clinical investigation (*42*–*44*). Tumor infiltrating lymphocytes (TILs), which can be found in tumors and in tumor-draining lymph nodes (tdLNs), recognize multiple tumor antigens and hold promise as therapeutics (*28*, *42*, *45*), but treatment with T cells alone is often not sufficient for overcoming the suppressive TME (*10*). We therefore evaluated whether cell-tethered IL-12 could enhance the antitumor efficacy of adoptively transferred tdLN-derived T cells in a preclinical mouse model. For this purpose, T cells from tdLN of BALB/c mice bearing subcutaneous CT26 colon cancer tumors were isolated and stimulated ex vivo with CD3/CD28 activation beads to generate a polyclonal population of CT26 tumor antigen–reactive T cells (Fig. 5A). This resulted in a cell product consisting of >90% T cells, >70% of which were central memory T cells as characterized by CD44 and CD62L coexpression (Fig. 5B).

The CD45-tethered IL-12 improved antitumor efficacy compared to adoptive transfer of T cells alone or in combination with native IL-12 (Fig. 5, C to F). This was consistent for mice bearing smaller or larger tumors at the time of adoptive transfer (30-mm^3^ versus 80-mm^3^ average starting tumor volumes). In mice bearing smaller tumors, cells tethered with IL-12 completely eradicated tumors in six of eight mice, and a seventh mouse experienced tumor regression followed by delayed tumor outgrowth compared to controls (Fig. 5, C and D). In contrast, only two of seven mice that had received T cells alone achieved complete tumor eradication, and only three of eight mice eradicated their tumors when the transferred T cells were coadministered with systemic IL-12 (Fig. 5D). All surviving mice rejected rechallenge with CT26 cancer cells, demonstrating durable immunity against the primary tumor (Fig. 5D). As observed above in the PMEL/B16 studies, the cell-tethered IL-12 did not cause overt toxicities in the form of weight loss (fig. S9). These results show that CD45-tethered IL-12 safely improves efficacy in a multitargeted tumor-specific T cell–adoptive transfer model.

### Cell-tethered IL-12 increases the activity of adoptively transferred T cells in the tumor

**Fig. 6.**
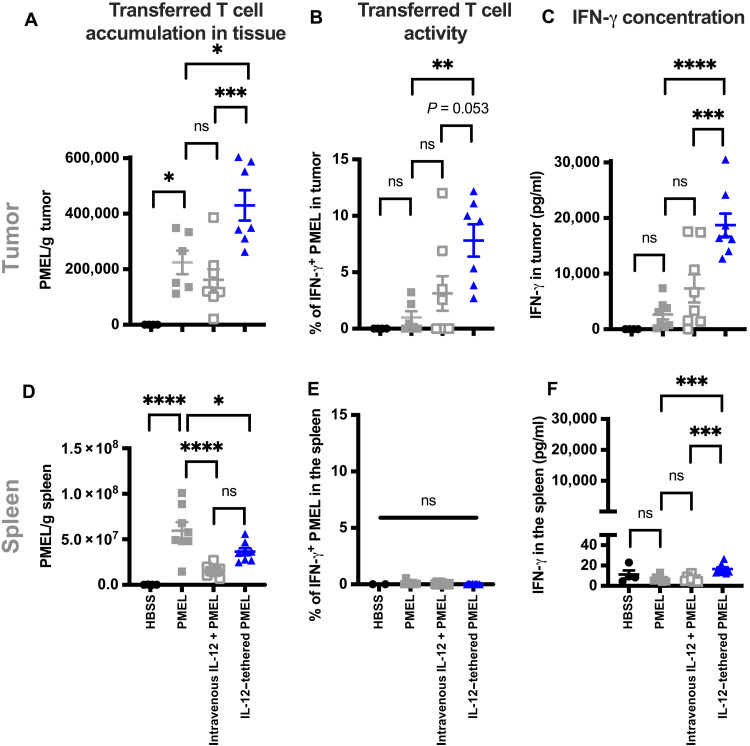
Tumor-focused effects of treatment with IL-12–tethered T cells. Comparison of in vivo infiltration and activity of 5 × 10^6^ PMEL T cells alone, tethered with CD45-targeted IL-12 (approximately 210 ng of IL-12) or in combination with intravenously coinjected IL-12 (500 ng) in tumor and nontumor tissues assessed 4 days after adoptive transfer. Numbers of adoptively transferred T cells per gram of tissue (**A** and **D**), frequency of adoptively transferred CD8 PMEL T cells expressing IFN-γ (**B** and **E**), and IFN-γ concentration in tumor (**C**) or spleen are shown (**F**). Data in (A), (B), (D), and (E) were generated by multicolor flow cytometry analysis; data in (C) and (F) were generated by Luminex cytokine analysis. All data were collected 4 days after T cell–adoptive transfer into mice bearing intradermal B16-F10 tumors. Statistical comparisons were made by one-way ANOVA followed by a Dunnett’s posttest to compare all groups to the PMEL treatment group. *P < 0.05, **P < 0.01, ***P < 0.001, and ****P < 0.0001.

To evaluate the ability of cell tethering to focus IL-12 activity to the TME, we compared immune activity in tumor and nontumor tissues. The IL-12–tethered PMEL T cells exhibited increased infiltration and IFN-γ production in tumor tissue compared to either adoptively transferred T cells alone or T cells coadministered with a 2.5-fold higher dose of systemic IL-12 (Fig. 6, A and B). This resulted in significant increases in both IFN-γ–positive PMEL T cells and IFN-γ concentration in the tumor compared to controls (Fig. 6C and fig. S10). In contrast to tumor tissue, increased PMEL T cell infiltration or IFN-γ production were not observed in the spleen (Fig. 6, D to F), a representative tissue in which the gp100 tumor antigen that is recognized by the PMEL T cells is not expressed. Similar to the immune cell profiling data, the IFN-γ concentration increased >1000-fold in tumors treated with IL-12–tethered T cells compared to the spleen (16.7 pg/ml in the spleen compared to 18,700 pg/ml in tumors; Fig. 6, C and F). Together, this shows that tumor-specific T cells tethered with IL-12 exhibit greater activation than achieved via coinjection of systemic IL-12 and drive immune activity preferentially in the tumor.

### IL-12–tethered T cells repolarize suppressive myeloid cells in the TME

**Fig. 7.**
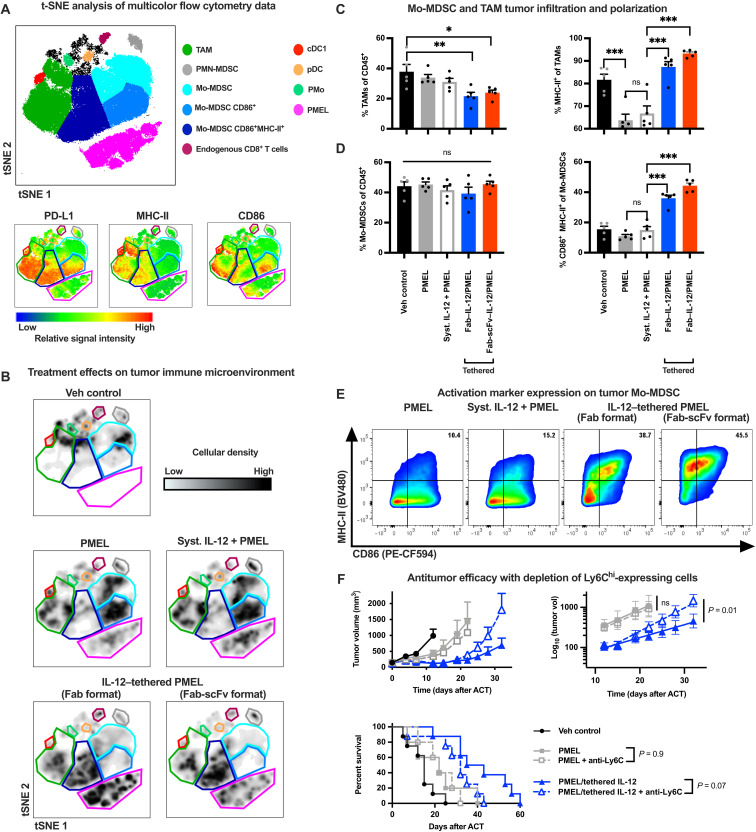
Adoptive transfer of IL-12–tethered T cells activates MDSCs and TAMs in the TME. (**A**) Analysis of immune cell types in B16-F10 tumors 4 days after treatment with 5 × 10^6^ PMEL T cells alone, coadministered with 500 ng of IL-12 iv or tethered with IL-12 before adoptive transfer (Fab or Fab-scFv anti-CD45 antibody formats) using multicolor flow cytometry, visualized by tSNE analysis. tSNE was based on five samples from each treatment group. Location of myeloid lineage cell types within tSNE space is shown. The gating strategy is shown in fig. S11. (**B**) Density plots showing the relative abundance of various cell subsets in tSNE space for individual treatment groups. Colored lines enclose cell populations of interest and match the colors in the legend to (A). (**C** and **D**) Relative proportion and activation marker expression on TAMs (C) and monocytic MDSCs (Mo-MDSCs) (D). (**E**) Representative plots comparing CD86 and MHC-II expression on Mo-MDSC across the various treatment groups. (**F**) Tumor growth inhibition by the indicated treatments with or without depletion of Ly6C^+^ cells. Data are plotted until two mice in a given group reach euthanasia criteria. Statistical comparisons in (C) and (D) are made by one-way ANOVA followed by a Tukey’s posttest to compare all groups to each other. Tumor growth inhibition was compared by a multivariate growth curve model of the exponential tumor growth phase (*68*). Survival was compared by a log-rank Mantel-Cox test. *P < 0.05, **P < 0.01, and ***P < 0.001. PMN, polymorphonuclear; pDC, plasmacytoid dendritic cell; pMO, patrolling monocyte.

To more broadly analyze the effects of IL-12–tethered T cells on the tumor immune microenvironment, we profiled myeloid lineage cells in the tumor using multicolor flow cytometry and analyzed the results using *t*-distributed stochastic neighbor embedding (tSNE; Fig. 7, A and B, and fig. S11). Monocytic MDSCs (Mo-MDSCs) and TAMs, two suppressive tumor immune cell types, were the most abundant myeloid lineage immune cell types present in B16-F10 tumors (Fig. 7B). This is consistent with other studies characterizing this as a suppressive, poorly immunogenic tumor background (*46*). Both the Fab and the higher-affinity Fab-scFv CD45-tethered IL-12 molecules reduced TAM infiltration and reversed suppression of MHC-II expression, an activation marker associated with a proinflammatory, antitumor M1-like macrophage phenotype (*47*, *48*), compared to treatment with PMEL T cells alone (Fig. 7C). The IL-12–tethered PMEL T cells did not affect the relative infiltration of Mo-MDSC (Fig. 7D) but did increase their surface expression of MHC-II and CD86 (Fig. 7, A and B), two activation markers involved in antigen presentation and T cell costimulation, respectively. This further resulted in significantly increased proportion of CD86/MHC-II double-positive tumor Mo-MDSCs compared to transfer of PMEL T cells alone (Fig. 7, D and E). Coadministration of systemic IL-12, by comparison, was ineffective at these dose levels (Fig. 7, D and E). Depletion of Mo-MDSCs with a depleting anti-Ly6C antibody diminished the antitumor efficacy of IL-12–tethered but not nontethered PMEL T cells (Fig. 7F). This suggests that the observed Mo-MDSC repolarization is at least partially required for the superior antitumor efficacy of IL-12–tethered T cells compared to nontethered controls. Together, these findings demonstrate that T cell–tethered IL-12 helps support immune activity in the TME by repolarizing suppressive myeloid immune cells into inflammatory effector phenotypes.

### Combination with immune checkpoint blockade further improves the antitumor efficacy of IL-12–tethered T cells

**Fig. 8.**
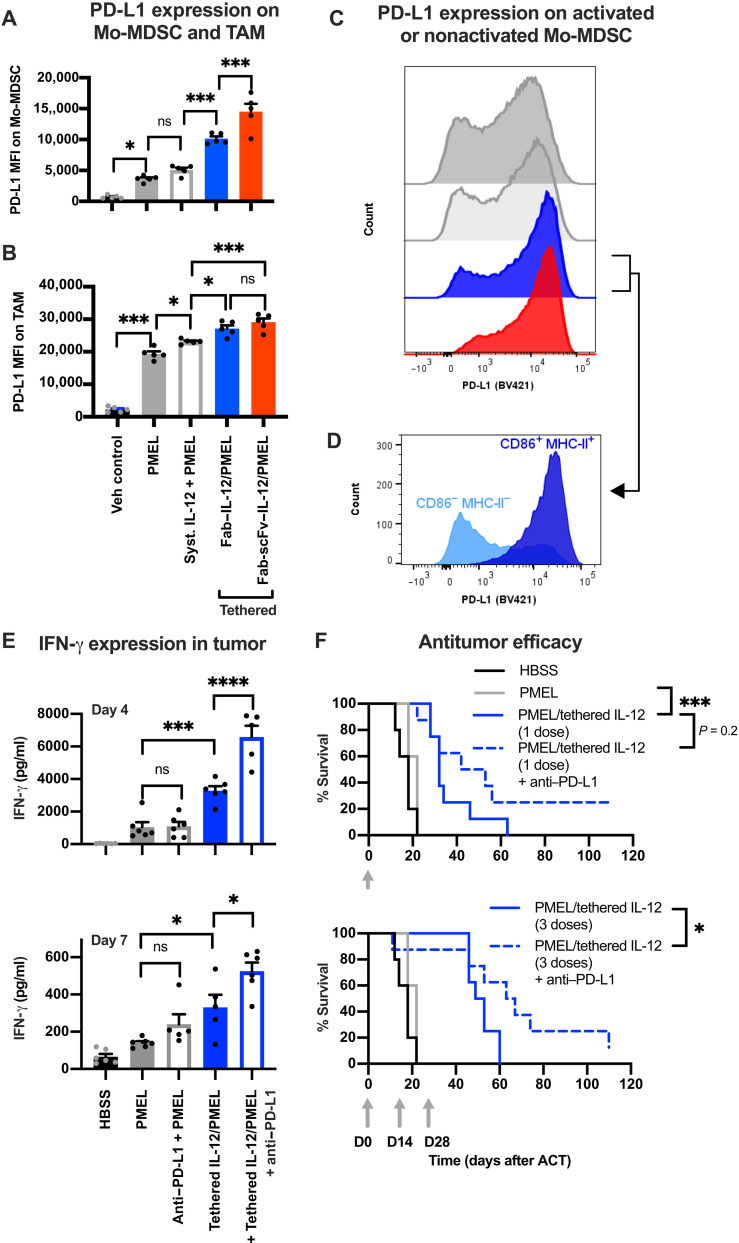
Improved activity and efficacy of IL-12–tethered T cells in combination with PD-L1 blockade. (**A** and **B**) PD-L1 expression on Mo-MDSC (A) and TAM (B) in tumors 4 days after treatment with 5 × 10^6^ PMEL T cells alone, coadministered with 500-ng, iv, IL-12, or tethered with IL-12 before adoptive transfer (Fab or Fab-scFv anti-CD45 antibody formats). Mo-MDSC are defined as CD45^+^/CD11b^+^/CD11c^−^/Ly6C^hi^/Ly6G^low^; TAM are defined as CD45^+^/CD11b^+^/CD11c^+^. (**C**) Representative PD-L1 histogram overlays of Mo-MDSC following the various treatment conditions; histogram color scheme matches (A) and (B) legend. (**D**) PD-L1 expression on repolarized (CD86^+^ MHC-II^+^) and nonrepolarized Mo-MDSC (CD86^−^ MHC-II^−^) following treatment with IL-12–tethered PMEL T cells (Fab anti-CD45 antibody format), demonstrating that PD-L1 is up-regulated on repolarized Mo-MDSC. (**E**) IFN-γ concentration in tumors 4 and 7 days after ACT in combination with biweekly administration of neutralizing antibody against PD-L1. (**F**) Antitumor efficacy from 5 × 10^6^ IL-12–tethered T cells in combination with PD-L1 blockade (biweekly administration for 6 weeks). Mice were treated with one (top) or three (bottom) doses of T cells, indicated by gray arrows; control groups are replotted on top and bottom plots for visual comparison. All studies were performed with C57BL6/J mice bearing subcutaneous B16-F10 tumors. Mice were preconditioned with cyclophosphamide 1 day before adoptive transfer; in the repeat dosing arms of (F), cyclophosphamide preconditioning preceded only the first cell therapy dose, and the second and third doses were administered in the absence of further preconditioning. Statistical comparisons of PD-L1 or IFN-γ expression were made by one-way ANOVA followed by a Tukey’s posttest to compare all groups to each other. Survival was compared by a log-rank Mantel-Cox test. *P < 0.05, **P < 0.01, ***P < 0.001, and ****P < 0.0001. D, day.

Immune activation in the tumor can also elicit negative feedback mechanisms, such as up-regulation of regulatory signaling molecules that suppress T cell activity (*49*). To assess this, we examined the expression of the inhibitory checkpoint protein programmed death ligand 1 (PD-L1) across tumor immune cells. Treatment with PMEL T cells increased PD-L1 expression across a range of tumor immune cells, including Mo-MDSCs, TAMs, and DCs (Fig. 8, A and B, and fig. S12A). Among these, tethered IL-12 further up-regulated PD-L1 expression on the Mo-MDSCs and TAMs (Fig. 8, A and B). Notably, within the Mo-MDSCs, up-regulation of PD-L1 expression was most prominent in the repolarized MHC-II/CD86 double-positive population (Fig. 8, C and D, and fig. S12B). To examine the mechanism of Mo-MDSC and TAM repolarization and PD-L1 up-regulation, we evaluated the effects of IL-12–tethered PMEL T cells in the presence of a neutralizing IFN-γ antibody. IFN-γ neutralization reduced the repolarization of Mo-MDSC and TAM and the up-regulation of PD-L1 induced by the IL-12–tethered T cells (fig. S13). Together, this demonstrates that both suppressive immune cell repolarization and negative regulatory feedback induced by IL-12–tethered T cells are at least partially mediated by the prominent up-regulation of IFN-γ expression in the tumor. This is consistent with previous studies demonstrating that IFN-γ not only induces pronounced antitumor effects but can also induce negative regulatory feedback (*50*, *51*).

The B16-F10 melanoma model is well established as a checkpoint refractory model in which PD-1 or PD-L1 blockade does not improve the overall survival alone (*52*, *53*) or in combination with PMEL T cell–adoptive transfer (*53*, *54*). However, based on the immune-activating TME effects induced by the IL-12–tethered T cells, coupled with negative regulatory feedback from also up-regulating PD-L1 expression on activated myeloid lineage cells in the tumor, we reasoned that checkpoint blockade may further enhance tumor immune activity and antitumor efficacy. PD-L1 blockade further increased IFN-γ expression in the tumor when combined with adoptive transfer of the IL-12–tethered cells but not when combined with PMEL T cells alone (Fig. 8E). The IL-12–tethered T cells also induced proliferation of endogenous CD8 T and NK cells in the tumor and additionally induced proliferation of endogenous CD4 T cells when combined with PD-L1 blockade (fig. S14). No endogenous CD4 T, CD8 T, or NK cell proliferation was induced by PMEL T cells alone or in combination with PD-L1 blockade (fig. S14). Together, this demonstrates that the tethered IL-12 can act on endogenous lymphocytes in the tumor, and this can be augmented by checkpoint blockade. These effects resulted in improved antitumor efficacy from the IL-12–tethered T cells, including an increased number of mice that had completely eradicated their tumors (Fig. 8F). Thus, combining checkpoint inhibitor therapy with IL-12–tethered T cell therapy can augment immune activity in the TME for further improved antitumor efficacy.

## DISCUSSION

Durable efficacy from ACTs in solid tumors remains a substantial unmet clinical need. One avenue to achieve this is by equipping the adoptively transferred T cells to overcome the immune-suppressive TME. Its potent and broad immune-activating properties make codelivery of IL-12 a promising approach. IL-12 has long been an attractive agent for cancer immunotherapy, but its use has been limited by high systemic toxicities (*29*). To safely harness the therapeutic potential of IL-12, it is critical to control IL-12 dosing and activity and to limit systemic effects from this potent immune stimulator.

The cytokine tethering approach described here is designed to provide defined cytokine dosages onto adoptively transferred cells. We demonstrated here that this safely enhanced T cell activity in three human and mouse T cell therapy models. Multiple cell surface receptors—including CD45, CD11a, and CD18—were able to facilitate tethering of IL-12 and IL-15 cytokines to T cells, and multiple different forms of CD45 tethering improved the antitumor efficacy of engrafted T cells more effectively than coadministration of the native cytokine. In the aggressive, immune-suppressed B16-F10 mouse melanoma model, IL-12 tethering strongly enhanced the antitumor efficacy of adoptively transferred tumor-specific PMEL T cells and focused IL-12 activity to the TME at doses in which systemic IL-12 coadministration was ineffective. IL-12 tethering also improved the activity of natural, nongenetically engineered T cells, including in vitro activity and cytotoxicity of human T cells generated by ex vivo DC priming and in vivo efficacy of mouse T cells expanded from tdLNs of CT26 tumor-bearing mice. Together, the present study demonstrates that directly tethering cytokines to the T cell surface by fusing to an antibody targeting highly expressed cell surface receptors safely harnesses the cytokine activity to improve the efficacy of ACT in both immune-suppressed (B16-F10 melanoma) and inflamed (CT26 colon carcinoma) tumor models.

Focusing IL-12 activity against the TME and avoiding systemic activity are believed to be critical to overcome its systemic toxicities (*31*). Previous approaches have used intratumoral delivery of IL-12 DNA by either electroporation (*55*) or viral delivery (*56*). Even cytokines delivered intratumorally, however, can leak into the vasculature to result in systemic toxicities (*57*). Drug-controlled IL-12 expression (*58*) or linking IL-12 to proteins that bind factors in the TME (*57*) hold potential to improve tumor retention following localized delivery. In the present study, tumor-specific T cells tethered with IL-12 delivered similar antitumor activity both when administered intravenously and intratumorally. Even when delivered systemically immune activation was much more pronounced in the TME than in nontumor tissues. In particular, treatment-induced IFN-γ concentrations were >1000-fold higher in the tumor compared to the spleen, for example. This suggests that presentation of the targeted antigens, which occurs in the TME and in tdLN but not in off-target tissues, is required for the pharmacodynamic effect of T cells tethered with the CD45-targeted IL-12. Our studies with MART-1–specific human T cells in vitro support this view: Tethered IL-12 only enhanced cytotoxicity and cytokine production against human melanoma cells expressing the MART-1 tumor antigen but not against melanoma cells that do not express MART-1. Together, this demonstrates that tethering IL-12 does not drive pronounced T cell responses on its own, but rather it enhances T cell activity upon TCR engagement of its cognate antigen. Tumor-specific antigen expression then focuses the tethered IL-12 function on the TME, avoiding systemic immune activation consistent with the absence of overt signs of toxicity in our mouse models.

Alternative approaches to couple cytokines with cell therapies include gene engineering to express the cytokine from the T cell. However, these approaches have proven toxic in human patients (*28*), likely due to poor expression control of IL-12. This approach also required daily media changes during cell therapy manufacturing to mitigate IL-12–induced suppression of ex vivo T cell expansion (*28*). Sustained exposure to IL-12 may drive T cell exhaustion (*59*), suggesting that approaches to transiently deliver IL-12 may be preferred over sustained, gene engineering–based approaches. The use of IL-12 mRNA to deliver transient IL-12 production for cell therapy has been explored, but, in preclinical models, this approach required intratumoral injection and combination with other immune-activating agents for durable antitumor efficacy (*60*). The transient nature of mRNA also complicates manufacturing: The mRNA would need to be delivered after cell manufacturing so that it is not lost during the manufacturing process, but mRNA for billions of cells, which are typical cell doses for solid tumor cell therapies in patients with cancer, would be cost-prohibitive. Nanoparticle-based approaches have been developed that facilitate transient cytokine delivery and are compatible with cell therapy manufacturing (*61*). These approaches, however, result in autocrine-focused cytokine function, which would limit the therapeutic benefit from cytokines such as IL-12. The surface-tethering approach described here is inherently transient and delivers the cytokine in a format that supports both autocrine and paracrine activity. Since the cytokine is tethered after cell manufacturing, it also avoids any potential negative effects of the cytokine on cells during manufacture and is also cost-effective even for tethering at least 100 s of billions of cells.

The paracrine function of the surface-tethered IL-12 supported pronounced activation of endogenous immune cells in the tumor. These were most prominent for Mo-MDSCs and TAMs, the two myeloid cell types that suppress CD8 T cell activity and are associated with resistance to immune checkpoint therapy (*62*–*64*). Systemically administered IL-12–tethered T cells repolarized Mo-MDSCs in the TME toward an antitumor, effector-like phenotype, while TAMs were converted to an M1-like effector phenotype characterized by high MHC-II expression. The T cell–tethered IL-12 was more effective at repolarizing each of these populations than a ~2.5-fold higher dose of coinjected cell-free IL-12. The tethered IL-12 also resulted in the up-regulation of PD-L1 on Mo-MDSCs and TAMs in an IFN-γ–dependent manner, consistent with previous reports for IFN-γ function (*51*). Negative feedback from the undesirable PD-L1 up-regulation was relieved by combination of IL-12–tethered T cells with PD-L1 blockade, which resulted in further increased tumor immune activity and improved antitumor efficacy. Together, this suggests that while the tethered IL-12 can help overcome immunotherapy resistance mechanisms by repolarizing suppressive tumor immune cells, in doing so, it also drives a PD-L1–based secondary resistance mechanism. Thus, IL-12–tethered T cell therapies may synergize with immune checkpoint therapies by each overcoming convergent resistance pathways.

Overall, the cell-tethering approach described here holds promise for safely harnessing the biology of potent immune modulators for cancer immunotherapy. Direct tethering to the T cell surface provides a regulated and controlled pharmacologic approach. This is expected to be critical for enabling the broad therapeutic utility of potent cytokines such as IL-12, IL-2, and IL-15, where poor dose control and systemic exposure causes unpredictable toxicity. The lack of gene engineering also facilitates the use of existing T cell therapy manufacturing processes, as the cytokines are tethered through a simple incubation after T cell manufacturing. Taken together, we believe that this cytokine tethering is a versatile approach for boosting the activity of T cell therapy candidates. Repertoire Immune Medicines has now opened a phase 1 clinical study to evaluate IL-12–tethered T cells in patients with solid tumor based on the scientific results described here.

## MATERIALS AND METHODS

### Cell lines

The MART-1–positive, HLA-A*02:01–positive human melanoma cell line SK-MEL-5; the MART-1–negative, HLA-A*02:01–positive human melanoma cell line A375; B16-F10 mouse melanoma cell line; Jurkat Clone E6-1 cell line; Jurkat CD45 knockdown cell line J45.0 l; and CT26 mouse colon cancer cell line were purchased from American Type Culture Collection and were maintained according to the manufacturer’s recommendations.

### Antibodies

For surface receptor profiling analyses, anti-human CD45 (clone 2D1), anti-human CD11a (clone Hi111), anti-human CD18 (clone CBR1FA1/2), anti-human CD2 (clones RPA2.1 and TS1/8), anti-human CD4 (clone SK3), anti-human CD8 (clones Hit8a and SK1), anti-human CD3 (clones Hit3a and UCHT1), anti-human IL-12Rβ1 (clone 69310), anti-human IL-12Rβ2 (clone s16020B), anti-human IL-7R (clone A019D5), anti-human IL-21R (clone 17A12), anti-human IL-2/IL-15Rβ (clone Tu27), anti-human common γ chain (clone TuGh4), and anti-human PD-1 (clones NaT105 and 2H7) were purchased from BioLegend. For surface-tethering analyses, anti-human (clone C11.5) and anti-mouse IL-12 (clone 15.6) were purchased from BioLegend, and anti-human IL-15 (clone 34559) was purchased from R&D Systems. Anti–phospho-STAT4 (p-STAT4) (pY693, clone 38) and anti–p-STAT5 (pY694, clone 47) were purchased from BD Biosciences. For in vivo studies, anti–IFN-γ (clone XMG1.2), anti–PD-L1 (clone 10F.9G2), and anti-Ly6C (clone Monts 1) were purchased from Bio X Cell. For PMEL-adoptive transfer studies and tumor immune microenvironment studies, antibodies are described in tables S1 and S2.

### Recombinant protein production

IL-15– and IL-12–tethered fusions and wild-type proteins were recombinantly produced from suspension-adapted human embryonic kidney–293 cells in serum-free media. All Fab antibody fragments were constructed using a human constant kappa and IgG1-CH1 domains. Proteins were affinity-purified using protein A (for IL-15/sushi-Fc fusion), KappaSelect or CH1 (for tethered fusions), or histidine affinity nickel–nitrilotriacetic acid (for native single-chain IL-12) resin, as appropriate (GE Healthcare), and then buffer exchanged into phosphate-buffered saline (PBS). All IL-15 protein used in this study (excluding IL-15–tethered fusions) comprised IL-15 noncovalently associated to the IL-15Rα sushi domain, which was genetically linked to the N terminus of a human Fc domain (IL-15/sushi-Fc). Approximate molecular weights were confirmed by reducing and nonreducing SDS–polyacrylamide gel electrophoresis, and aggregation or multimeric status was determined by size exclusion chromatography (SEC). On the basis of aggregation or multimeric status, proteins were purified by SEC using a HiLoad Superdex 200 prep grade columns (GE Life Sciences) on an ÄKTA Pure chromatography system (GE Life Sciences).

### Human T cell activation using CD3 and CD28 stimulation

Primary human T cells from healthy donors were obtained from BioIVT (Colmar, PA) and were activated by Human T-Activator Dynabeads (Thermo Fisher Scientific Inc.) according to the manufacturer’s instructions and in the presence of human IL-2 (10 ng/ml) (R&D Systems) in complete T cell media (RPMI 1640 media containing 10% fetal bovine serum and 1% GlutaMAX) according to the manufacturer’s instructions. After 3 days of stimulation, the anti-CD3/CD28 beads were removed, and cells were maintained in IL-2 containing human T cell complete media [IL-2 (20 ng/ml)] for up to 4 days before experimentation.

### Receptor surface expression analysis

Relative receptor expression levels were determined using fluorescent-labeled antibodies and fluorescent bead standards. For accurate receptor quantitation, it was necessary to ensure that the receptor antibodies were used at concentrations that saturate the cell surface receptor (rather than simply mediate a positive binding signal). Alexa Fluor 647–labeled antibodies used for receptor quantitation (table S1) were titrated to determine the appropriate antibody dilution for receptor quantitation. Briefly, antibodies were serially diluted into staining buffer [PBS (pH 7.4) and 0.1% bovine serum albumin (BSA)] and incubated with human total CD3 T cells at 4°C for 30 min in the dark. Cells were washed, resuspended into staining buffer, and analyzed on a FACSCelesta flow cytometer using FACSDiva software (BD Biosciences). Dead cells were excluded from analysis by using Zombie Aqua viability dye (BioLegend).

To quantitate antibody binding levels, the fluorescent antibodies were incubated at concentrations determined above to result in saturating binding. A standard curve was generated using fluorescent bead standards (Quantum MESF Alexa 647 Beads, Bangs Laboratories Inc.) to correlate fluorochrome median fluorescent intensity (MFI) to the number of fluorochromes. Fluorochromes per cell were then converted to antibodies per cell based on antibody degree of labeling provided by the manufacturer. While most antibodies resulted in fluorescent intensities within the standard curve range, anti-CD45 and anti-CD8 antibodies resulted in MFI above the standard curve, indicating a greater number of fluorochromes per cell than the number of fluorochromes in the highest bead standard. To facilitate accurate quantitation, MFI was reduced by diluting the fluorescent antibody into a solution containing the same clone of antibody but without the fluorescent label. This would maintain saturating binding of the cell surface receptor, but only a fraction of the bound antibodies would be fluorescent, thereby reducing the MFI into the standard curve range. The labeled versus nonlabel antibody ratios were then factored into the calculation of total antibodies bound per cell.

### Tethered fusion cellular binding analysis

For tethered fusion binding analysis, serial dilutions of tethered fusions were incubated with human T cells for 1 hour in T cell media at 37°C. Unbound tethered fusion was removed by washing two times with T cell media. Cell-loaded cytokine was analyzed using flow cytometry by staining cells with anti–IL-15 or anti–IL-12 antibodies as appropriate. Dead cells were excluded from analysis using a viability dye such as Zombie Aqua or Zombie Violet (BioLegend).

### Pulse bioassay

Human T cells (Biological Specialty Company) were activated as described above. Activated T cells were incubated with serial dilutions of tethered fusions for 1 hour at 37°C. Nonbound tethered fusion was removed by washing three times in full T cell media. Cells were then plated at 0.5 × 10^6^ cells/ml in 96-well plates. Controls evaluating only the antibody portion of the tethered fusion, competition with soluble antibody competitor, or pulse incubation of cells with free cytokine were included where indicated. T cell expansion was analyzed using CellTiter-Blue Cell Viability Assay (Promega Corp.) according to the manufacturer’s instructions.

### STAT4 and STAT5 phosphorylation analysis

Following incubation with IL-12 or IL-15 constructs, cells were analyzed for activation of intracellular signaling by assessing STAT4 or STAT5 phosphorylation, respectively. Cells were fixed by incubation in 1.5% paraformaldehyde at room temperature for 10 min. Cells were then pelleted by centrifugation, and supernatants were aspirated and permeabilized in ice-cold methanol for 10 min at 4°C. The cells were then pelleted by centrifugation and washed twice with a blocking buffer (PBS containing 1% BSA). Fixed and permeabilized T cells were then stained with fluorescent labeled anti–p-STAT4 antibody or anti–p-STAT5 antibody diluted 1:50 in blocking buffer for 20 min at room temperature. The p-STAT4 antibody was raised against phosphorylated human STAT4 (pY693) but cross-reacts with phosphorylated mouse STAT4 based on sequence identity and was therefore used on both human and mouse T cells. Antibody-stained cells were then washed two times with blocking buffer for 5 min each at room temperature, resuspended, and analyzed on a FACSCelesta flow cytometer using FACSDiva software (BD Biosciences).

### In vitro cytotoxicity and cytokine response analysis

Cytotoxicity of MART-1–specific T cells was assessed against partially HLA-matched (HLA-A*02:01–positive) cancer cells lines that express (SK-MEL-5 cell line) or do not express (A375 cell line) the MART-1 antigen. MART-1–specific T cells were first generated from an HLA-A*02:01 human donor by stimulating human T cells with autologous mDCs following previously described approaches (*65*, *66*). Briefly, matured mDCs were incubated with an immunodominant MART-1 antigen (ELAGIGILTV; New England Peptide) and then cocultured with autologous human T cells. Cryopreserved mDCs were thawed and peptide pulsed for a second round of T cell priming and expansion.

MART-1–specific T cells were then assessed for cytokine production and cytotoxicity following coincubation with SK-MEL-5 or A375 cancer cells. Briefly, T cells were incubated with 0 to 160 nM CD45-tethered IL-12 for 30 min at 37°C, and then unbound IL-12 constructs were removed by washing. T cells (“effectors”) were then cocultured with cancer cells (“targets”) at a range of effector:target ratios in T cell media (5% human AB serum and 1% GlutaMAX; CTS AIM-V SFM, Thermo Fisher Scientific). Following 3- to 4-day coincubation, supernatants were removed, clarified by centrifugation, and stored at −80°C. Residual T cells were recovered by gently washing, and cancer cell survival was analyzed by MTT assay (Sigma-Aldrich) following the manufacturer’s instructions. Controls consisting of target cells alone or target cells treated with 10% dimethyl sulfoxide at experiment end point were used to define 0 and 100% killing. Supernatants were analyzed using the Human IFN-γ Quantikine ELISA Kit (R&D Systems) according to the manufacturer’s instructions. The presence of T cells specific for the MART-1 antigen was assessed by flow cytometry using fluorescent-labeled MHC tetramers presenting the MART-1 antigen ELAGIGILTV (MBL International).

### Quantification of IL-12 loading by untethering assay

To assess the amount of tethered IL-12 loaded onto the T cells, the IL-12 was dissociated using a low pH wash step and analyzed by enzyme-linked immunosorbent assay (ELISA). Briefly, cells were pelleted by centrifugation, and the cell-tethered IL-12 was dissociated with a 30-min incubation in ice-cold untethering buffer [100 mM glycine, 150 mM NaCl, and 0.3% BSA (pH 2.5)]. The solution was adjusted to pH 5.5 by addition of 3 M sodium acetate, aliquoted, flash-frozen in liquid nitrogen, and stored at −80°C. Samples were quantitated using a custom ELISA specific for the anti-CD45/IL-12 fusion. Briefly, 96-well flat-bottom plates (Nunc Maxisorp) were coated with recombinant human CD45 (R&D Systems) by overnight incubation at 4°C. The solution was then removed, and wells were washed three times with PBS containing 0.05% Tween 20 detergent (PBST) using a BioTek 405 LS plate washer. Wells were then blocked with blocking buffer (Sword Blocker, Sword Diagnostics, Chicago, IL) for at least 1 hour at room temperature. The blocking buffer was then removed, and the wells were washed three times with PBST. The untethered IL-12 samples were diluted to the linear range of the assay (typically 1:1000 to 1:4000 dilution) using Sword Diluent SDI-802 (Sword Diagnostics), added to the ELISA plate, and incubated for 2 hours at room temperature. Plates were washed three times with PBST, stained with a biotinylated anti–IL-12 detection antibody (R&D Systems, clone BAF219) in Sword Diluent SDI-815 (Sword Diagnostics), and incubated for 1 hour at room temperature with gentle shaking. Wells were washed three times using PBST, stained for 30 min at room temperature with horseradish peroxidase–conjugated streptavidin (R&D Systems), and washed three times, and then the signal was “boosted” with a 15-min incubation at room temperature using Sword Signal Booster. Last, Sword Development solution was added to each well, incubated in the dark for 30 min, and analyzed for fluorescence emission at 700 nm upon excitation at 530 nm on a Tecan M200 plate reader. A standard curve generated from four-parameter logistic regression (using Graphpad Prism) on serially diluted standards of CD45-targeted IL-12 was used to calculate sample concentrations.

### Mouse experiments

For experiments performed at Repertoire Immune Medicines, TCR transgenic pmel-1 mice [B6.Cg-Thy1a/Cy Tg(TcraTcrb)8Rest/J] and wild-type C57BL/6 mice were purchased from the Jackson Laboratory. Mice were housed either in the Charles River Accelerator and Development Lab (CRADL) or Mispro Biotech Services animal facilities. All animal procedures were performed in accordance with the National Institutes of Health Guide for the Care and Use of Laboratory Animals and were approved by the CRADL or Mispro Institutional Animal Care and Use Committee, respectively. For experiments performed at the Technical University of Denmark, pmel-1 mice were purchased from the Jackson Laboratory and subsequently bred at the Department of Experimental Medicine, University of Copenhagen. Wild-type C57BL/6JRj and BALB/cJRj mice were purchased from Janvier Labs (and housed at the Department of Experimental Medicine, University of Copenhagen). All experimental procedures were approved by the Danish National Animal Experiment Inspectorate and the Institutional Ethics Review Board.

### Mouse T cell activation and expansion

For CD8 PMEL T cell isolation and activation, CD8 T cells were isolated from the spleens and LNs of pmel-1 TCR transgenic mice [B6.Cg-Thy1a/Cy Tg(TcraTcrb)8Rest/J; the Jackson Laboratory]. Briefly, spleens and LNs were processed with a GentleMACS Octo Dissociator (Miltenyi Biotec) and passed through a 70-μm strainer. Cells were washed by centrifugation, and CD8 T cells were purified using a CD8a T cell isolation kit and a LS column and separator following the manufacturer’s protocol (Miltenyi Biotec). The isolated CD8 PMEL T cells were cryopreserved in CryoStor CS10 media (STEMCELL Technologies).

For adoptive cell therapy studies, PMEL T cells were activated and expanded by CD3/CD28 as described in previous protocols (*67*). Cells were harvested, washed in PMEL media, and used for adoptive cell therapy studies either alone or following loading with mouse CD45-tethered IL-12. For the polyclonal T cell–adoptive transfer studies, the tdLN of CT26 tumor-bearing mice were harvested 6 to 7 days following tumor inoculation. To obtain a single-cell suspension, the tdLNs were minced and then dispersed by filtering through a 70-μm strainer, centrifuged, and washed. Cells were then activated and expanded as described for PMEL T cells above.

### Adoptive cell therapy studies

For adoptive cell therapy studies in the PMEL/B16-F10 model, 4 × 10^5^ to 10 × 10^5^ B16-F10 melanoma cells were inoculated intradermally or subcutaneously (as indicated per study) into the shaved right flanks of 9- to 12-week-old female C57BL/6 mice. CD8 PMEL T cells were activated and expanded as described above. Activated CD8 PMEL T cells were incubated with vehicle control or 125 to 160 nM CD45-tethered IL-12 for 30 min 37°C, washed three times [once with PMEL media and twice with Hanks’ balanced salt solution (HBSS)], and resuspended in HBSS. All PMEL studies use the Fab format of CD45-tethered IL-12 unless otherwise indicated. One day before adoptive transfer (designated as day −1), mice were lymphodepleted with intraperitoneal injection of cyclophosphamide (Sigma-Aldrich) at 4 mg per mouse. Tumor dimensions were monitored by caliper measurements, and tumor volume was calculated as length × width^2^ × 0.5. T cells were administered by intravenous injection unless otherwise indicated. Systemic IL-12 was administered by intravenous injection within 2 hours of adoptive cell transfer. For IFN-γ neutralization studies, at the day of ACT therapy and 2 days after, mice were injected intraperitoneally with 300 μg of anti–IFN-γ antibody (Bio X Cell) in 100 μl of HBSS. For combination with checkpoint blockade, anti–PD-L1 antibody (Bio X Cell) was administered at a dose of 200 μg per mouse twice weekly. For depletion of Ly6C^+^ cells, anti-Ly6C (Bio X Cell) was injected intraperitoneally at a dose of 300 μg per mouse twice weekly.

For cell therapy studies in the CT26 model, 3 × 10^5^ CT26 colon cancer cells were inoculated subcutaneously 10 or 11 days before ACT. Reactivated and expanded T cells isolated from tdLNs of CT26 tumor-bearing mice (prepared as described above) were incubated with vehicle control or CD45-tethered IL-12 as described for PMEL T cells above, washed, and then dosed by intravenous administration (without lymphodepletion before ACT).

### Tissue collection and analysis

Blood was collected into EDTA-coated whole-blood collection tubes. For cytokine analysis, blood was separated by centrifugation (1000*g*, 10 min), and plasma supernatants were stored at −80°C. Plasma cytokine levels were analyzed using a ProcartaPlex Luminex kit (Thermo Fisher Scientific) and a Bio-Plex 200 instrument (Luminex Corp). For flow cytometry analysis, red blood cells (RBCs) were lysed using a hypotonic buffer and washed three times. CountBright Absolute flow cytometry counting beads (Thermo Fisher Scientific) were added to the blood during the RBC lysis step. Cells were then washed in staining buffer (PBS containing 0.5% BSA and 2 mM EDTA) and resuspended in a master mix containing the staining antibodies. The antibody mixture was incubated for 10 min at room temperature and protected from light. Cells were washed in staining buffer two times and analyzed by flow cytometry on a FACSCelesta, and data were analyzed in FlowJo (Becton Dickinson).

For cytokine analysis in tissues, spleens and tumors were weighed and then incubated in PBS containing 0.1% Tween 20 (Fisher BioReagents) and 1× Halt Protease Inhibitor Cocktail (Thermo Fisher Scientific) at 37°C for 30 min. Tissue was then centrifuged, and supernatants were collected and stored at −80°C before analysis of IFN-γ expression by Luminex (Thermo Fisher Scientific). For flow cytometry analysis of tissues, tissues were excised, weighed, and processed to single-cell suspensions. Tumors were minced with scalpels, digested in tumor dissociation enzyme mix for murine tumors (Miltenyi Biotec) in a shaking water bath at 37°C for 40 min, and mashed through 70-μm cell strainers. Spleens were mashed through 70-μm cell strainers. Cells were then washed in staining buffer followed by resuspension in staining buffer containing appropriate antibodies for 10 min at room temperature or 30 min on ice and protected from light. Cells were then washed and resuspended in staining buffer for analysis by flow cytometry and acquired on a FACSCelesta or on LSRFortessa X-20 (Becton Dickinson) using FACSDiva and further analyzed in FlowJo. For tissues analyzed for IFN-γ expression by intracellular cytokine staining by flow cytometry, GolgiStop Protein Transport Inhibitor was added to the solution during processing to single-cell suspensions. For intracellular staining, following staining of cell surface receptors, the cells were washed three times in staining buffer, resuspended in Fixation/Permeabilization Solution (Thermo Fisher Scientific), and incubated overnight (4°C). The next day, samples were centrifuged and washed three times in permeabilization buffer. Antibodies for IFN-γ were incubated with the permeabilized cells for 30 min at room temperature protected from light and then washed two times in staining buffer and analyzed by flow cytometry on a FACSCelesta, and data were analyzed in FlowJo. tSNE clustering analysis was performed in FlowJo using the built-in downsample and tSNE tools. Samples (*n* = 5 per group) were downsampled to 10,000 viable CD45^+^ cells, concatenated, and subjected to tSNE analysis based on all fluorescent parameters except viability dye and CD45 with opt-SNE set to a maximum of 1000 iterations, 100 perplexity, and the Barnes-Hut algorithm. Overall, tSNE heatmaps are based on the complete set of samples. Cellular density plots are based on the total cells for each group and thereby display the average within groups.

### Statistical analysis

For comparisons between two samples, statistical significance was evaluated using Student’s *t* test. For multiple comparisons, statistical significance was evaluated using analysis of variance (ANOVA), followed by Dunnett’s or Tukey’s posttest to compare all groups against a single control or all groups against each other, respectively. Survival comparisons were made by a log-rank Mantel-Cox test. Graphs and statistical analyses were performed using Prism (GraphPad Software). A *P* < 0.05 was considered statistically significant. Data are reported as means ± SEM unless otherwise indicated.

## Notes

View/request a protocol for this paper from *Bio-protocol*.
