# Supplementary material for: Cell surface–tethered IL-12 repolarizes the tumor immune microenvironment to enhance the efficacy of adoptive T cell therapy
Source: Sci Adv. 2022 Apr 27;8(17):eabi8075. doi: 10.1126/sciadv.abi8075 (PMC9045725; doi:10.1126/sciadv.abi8075)
Supplement: Supplementary file 1 — Figs. S1 to S14 Tables S1 and S2 [file sciadv.abi8075_sm.pdf]

Science Advances
AAAS

Supplementary Materials for

Cell surface–tethered IL-12 repolarizes the tumor immune microenvironment to enhance the efficacy of adoptive T cell therapy

Douglas S. Jones II, Jonathan D. Nardozzi, Katharine L. Sackton, Gulzar Ahmad, Esben Christensen, Lars Ringgaard, De-Kuan Chang, Ditte E. Jaehger, Jacob V. Konakondla, Martin Wiinberg, Kate L. Stokes, Alvin Pratama, Karsten Sauer, Thomas L. Andresen*

*Corresponding author. Email: tlan@dtu.dk

Published 27 April 2022, *Sci. Adv.* **8**, eabi8075 (2022)
DOI: 10.1126/sciadv.abi8075

This PDF file includes:

Figs. S1 to S14
Tables S1 and S2

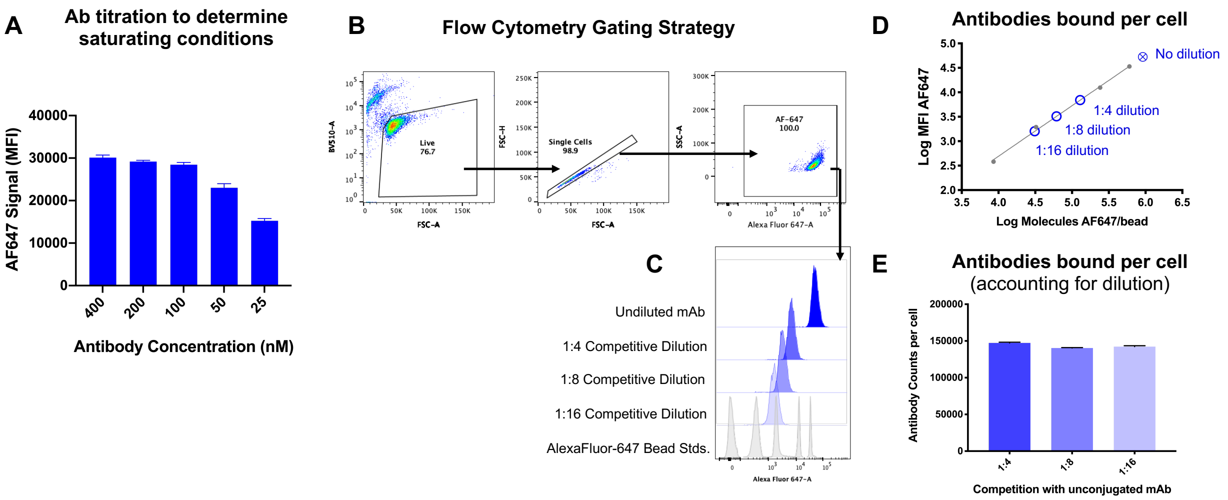

Figure S1. Receptor quantification strategy. (A) Example antibody titration (with CD45 antibody) on T cells to determine antibody concentration required for saturating binding. **(B)** Representative flow cytometry gating strategy for receptor quantification (for CD45 antibody). Live Cell (Zombie Aqua, BV-510 negative), Singlets (FSC-H vs FSC-A), and $CD45^+$ cell gate (AF-647-positive). **(C)** Histogram of median fluorescence intensity (MFI) of undiluted AF-647-conjugated CD45 mAb, compared to samples competitively diluted with unconjugated CD45 mAb to reduce MFI to a signal within linear range of AF-647 bead standard curve. For serial dilution the antibody concentration remains fixed at the saturating concentration but the ratio of labeled:unlabeled antibody decreases and correspondingly decreases MFI into standard curve range. **(D)** Conversion of histogram MFI values in panel (C) to number of antibodies per cell using microbead standard curve. Fold dilution with unconjugated antibody is noted. **(E)** Quantification of CD45 antibodies per cell from the AF-647 bead standard curve, accounting for competitive dilution with unconjugated anti-CD45 mAb.

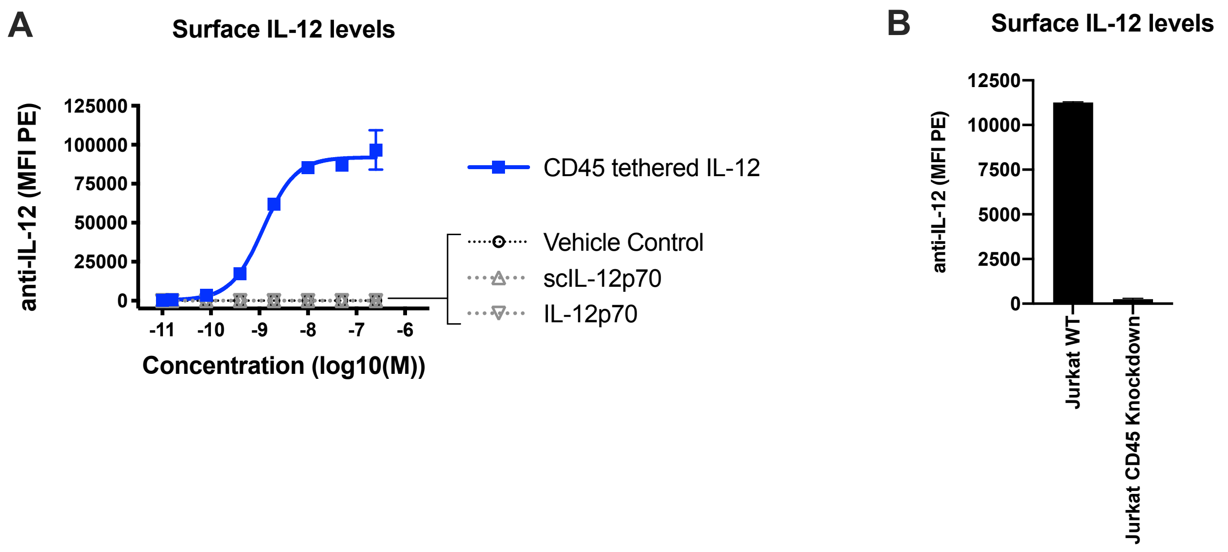

Figure S2. Cellular loading is driven by antibody targeting moiety. (A) Cellular loading comparison of human CD45-tethered IL-12, native IL-12 (IL-12p70) or single-chain IL-12 (scIL-12p70) onto human T cells assessed via anti-IL-12 staining by flow cytometry. **(B)** Loading of human CD45-tethered IL-12 onto wild-type or CD45 knock-down Jurkat T cells is assessed via anti-IL-12 staining by flow cytometry. Error bars represent SD; error bars smaller than the size of the symbol may not be visible.

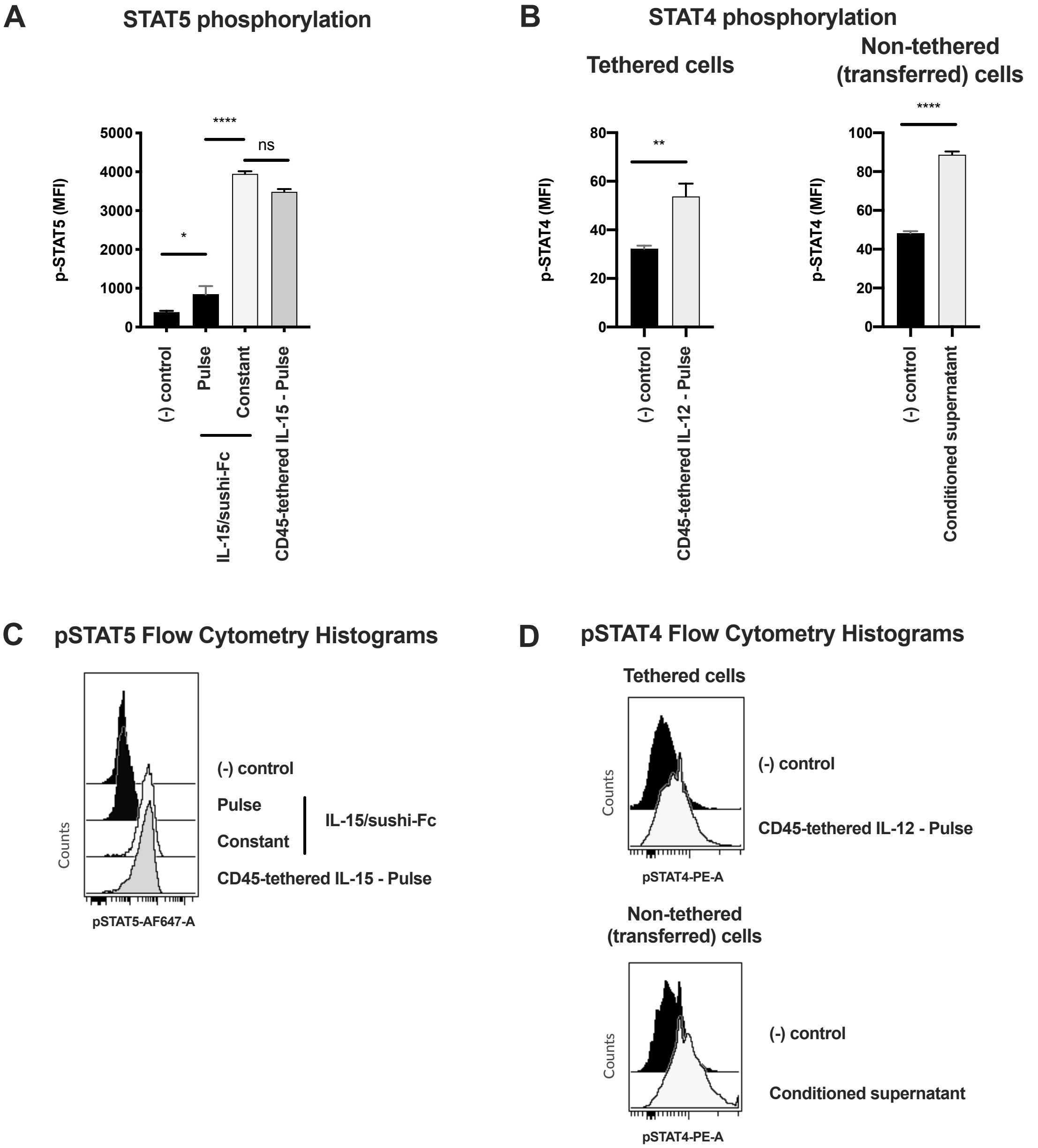

Figure S3. Intracellular signaling induced by CD45-tethered cytokines. (A) Intracellular signaling activity downstream of IL-15 receptors was assessed by staining for STAT5 phosphorylation 1 day after pulse incubation of IL-15/sushi-Fc cytokine or CD45-tethered IL-15, in which unbound cytokine was removed by washing prior to plating in culture. Activity from constant incubation with a saturating amount of IL-15/sushi-Fc is included as a positive control. **(B)** Intracellular signaling activity downstream of IL-12 receptors was assessed by staining for STAT4 phosphorylation 1 day after pulse incubation (left). One day after pulse incubation, non CD45-IL-12 tethered T cells were treated with conditioned supernatant from tethered cells for 30 min prior to staining for STAT4 phosphorylation to evaluate ability for the tethered IL-12 to transfer to non-loaded cells (right). **(C-D)** Representative phospho-STAT flow cytometry histograms for panels A and B are shown in C and D, respectively..

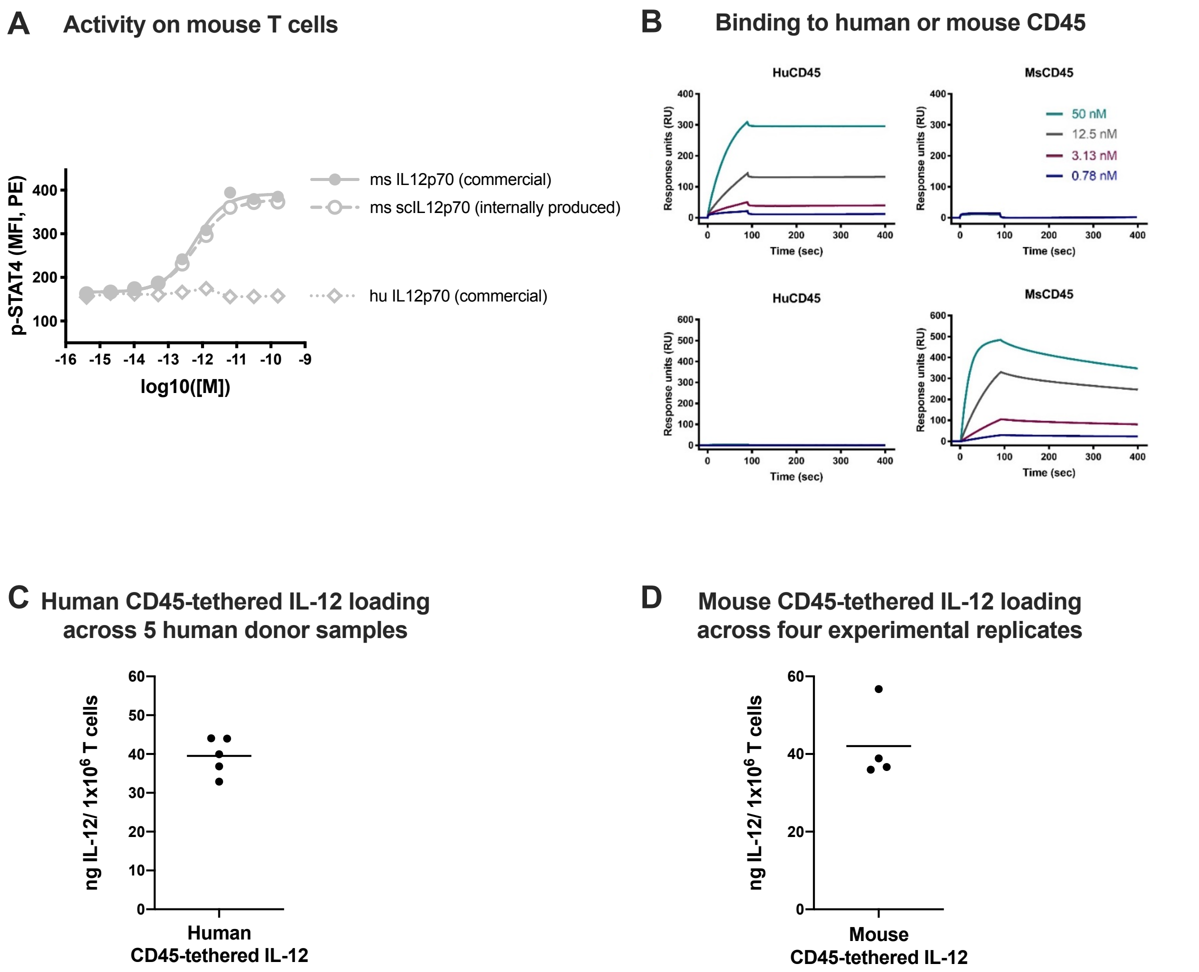

Figure S4. Loading quantification and cross-reactivity of IL-12 and tethering antibodies. **(A)** Phosphorylation of STAT4 on mouse T cells in response to 30 minute incubation with serial dilutions of various human or mouse IL-12 constructs – single chain mouse IL-12, commercial recombinant mouse IL-12 (R&D Systems), or commercial recombinant human IL-12 (R&D Systems); demonstrates human IL-12 is inactive on mouse T cells. **(B)** Binding curves measured by surface plasmon resonance for human (top) and mouse (bottom) CD45-tethered IL-12 to both human and mouse CD45; demonstrates human CD45-tethered IL-12 does not bind mouse CD45. **(C-D)** Loading comparison of (C) human CD45-tethered IL-12, comprising anti-human CD45 Fab antibody clone h9.4 and human single-chain IL-12, onto human T cells and of (D) mouse CD45-tethered IL-12, comprising anti-mouse CD45 Fab antibody clone M1/9.3.4.HL.2 and mouse single-chain IL-12, onto mouse PMEL T cells;, demonstrating similar degree of loading for the mouse and human CD45-tethered IL-12 constructs. In each case loading was assessed by dissociating the CD45-tethered IL-12 from the cells by a low pH wash (pH = 2.5) followed by quantification using a human or mouse CD45-tethered IL-12-specific ELISA, as appropriate. Each data point represents average loading from an experimental replicate performed on a separate day (n=4 loading experiments for mouse, n=5 loading experiments for human, with each replicate comprising a different human donor).

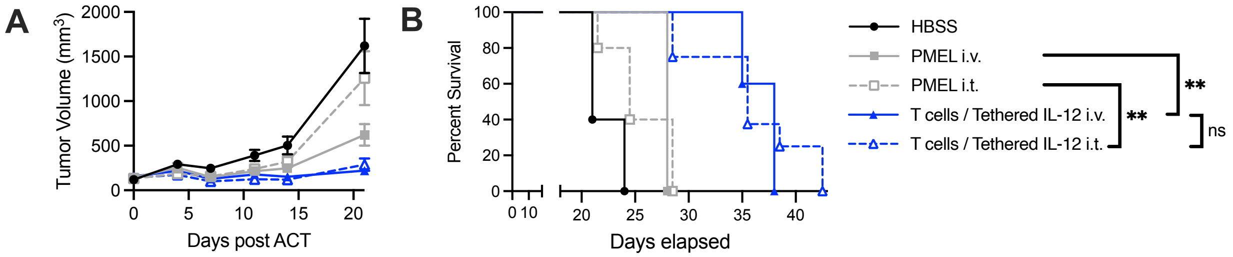

Figure S5. Comparison of anti-tumor efficacy by i.v. or i.t. administration. (A-B) Anti-tumor efficacy assessed by tumor growth inhibition (A) or overall survival (B) following treatment of C57BL6/J mice bearing subcutaneous B16-F10 tumors with 5x10^6 PMEL T cells alone or loaded with CD45-tethered IL-12, administered i.v. or i.t. as indicated. Survival was compared by a log-rank Mantel-Cox test. *$**P<0.01$*.

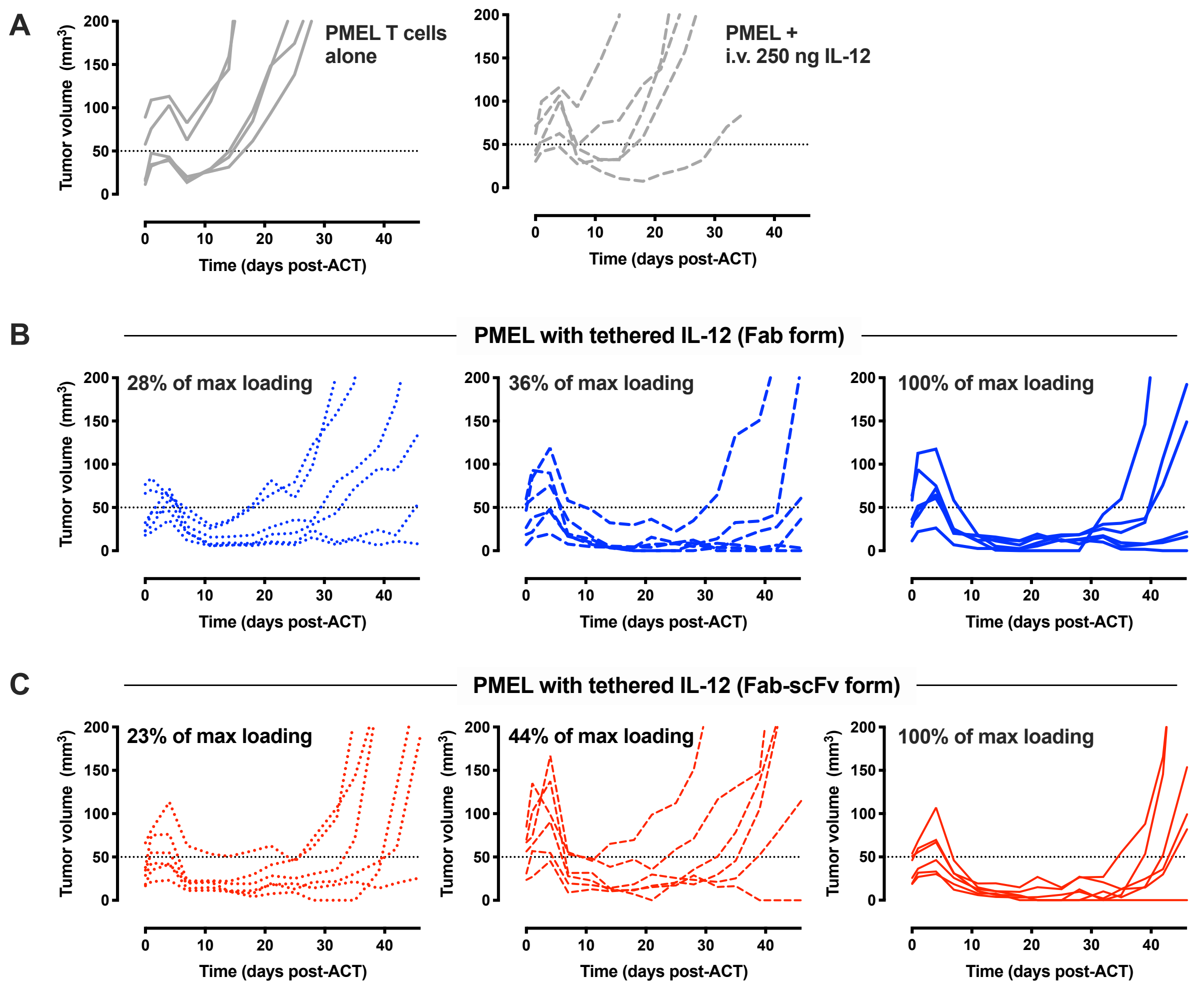

Figure S6. Tumor growth curves from individual mice treated with two different forms of CD45-tethered IL-12. (A-C) Tumor growth curves in individual mice bearing intradermal B16-F10 tumors following treatment with (**A**) PMEL T cells alone (n=5) or in combination with 250 ng systemic IL-12 (n=5), (**B**) PMEL T cells tethered with increasing amounts of the Fab format of CD45-tethered IL-12 (n=6), or (**C**) PMEL T cells tethered with increasing amounts of the Fab-scFv format of CD45-tethered IL-12 (n=6). PMEL T cell dose is 5×10^6 cells for all conditions.

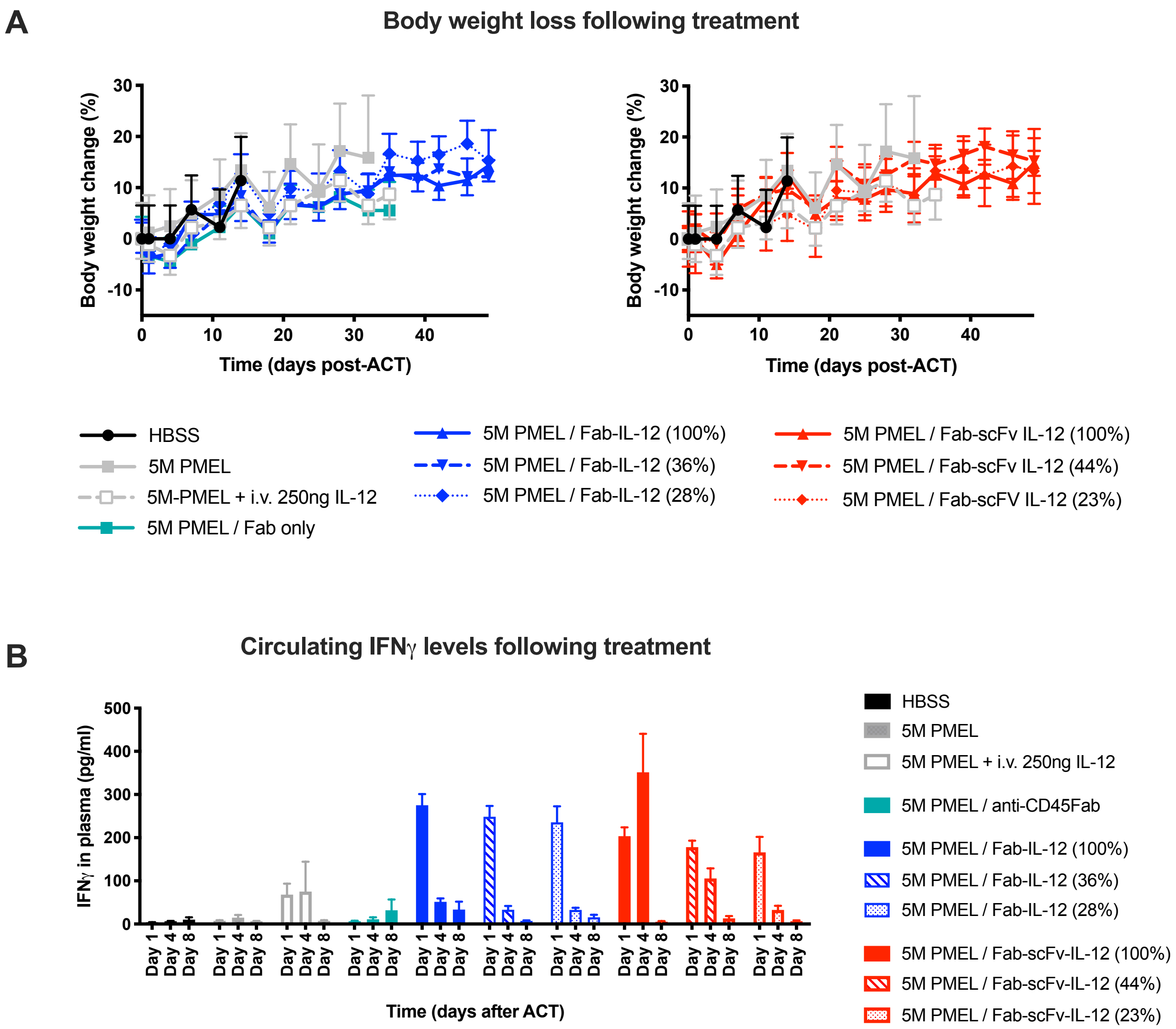

Figure S7. Systemic profile from IL-12-tethered PMEL T cell treatment. (A) No overt toxicities observed in the form of body weight loss following injection of PMEL T cells tethered with Fab (left) or Fab-scFv (right) forms of CD45-tethered IL-12. Control treatment conditions are replotted on both left and right plots for reference. **(B)** Circulating IFNγ concentrations 1, 4, and 8 days after adoptive transfer.

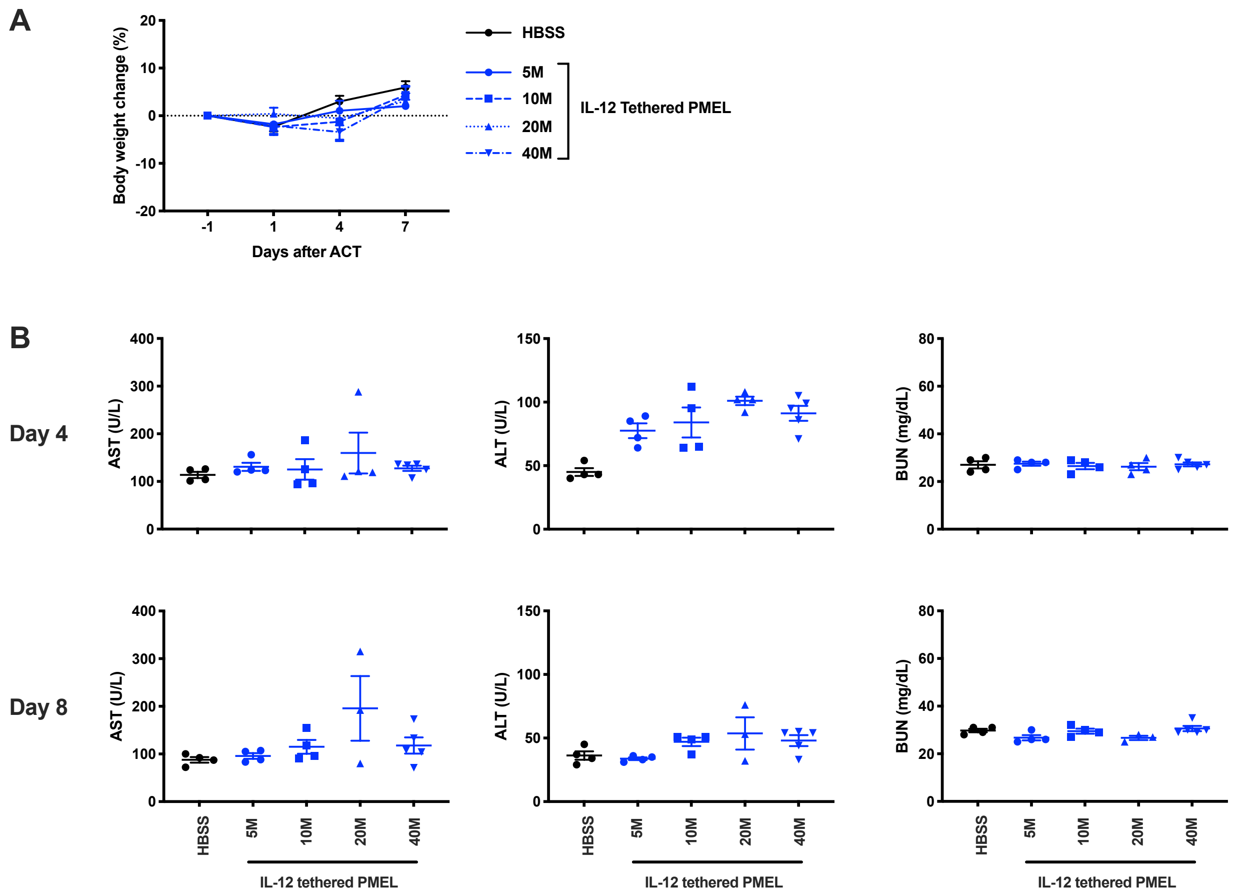

Figure S8. Safety profile from escalating doses of IL-12-tethered PMEL T cells. (A) Body weight loss with dose escalation up to 40 × 10^6 PMEL T cells loaded with CD45-tethered IL-12 (Fab format). PMEL T cells in this study were administered in absence of lymphodepleting preconditioning. **(B)** Serum analysis of liver (alanine aminotransferase and aspartate aminotransferase) and kidney (BUN) toxicology biomarkers 4 and 8 days after dose escalation adoptive transfer as in (A). ALT: alanine aminotransferase, AST: aspartate aminotransferase, BUN: blood urea nitrogen.

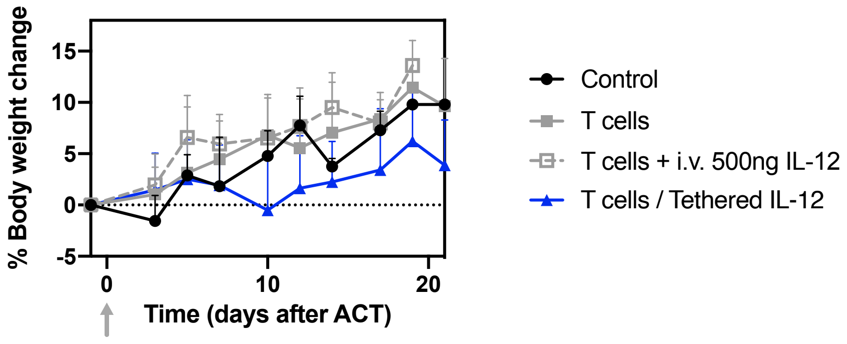

Figure S9. Body weight loss from IL-12 tethered to endogenous, multitargeted T cells. No overt toxicities in the form of body weight loss following treatment of CT26 tumor-bearing Balb/c mice with T cells expanded from tumor-draining lymph nodes of CT26 tumor-bearing mice alone, in combination with 500 ng i.v. IL-12, or loaded with CD45-tethered IL-12 prior to adoptive transfer.

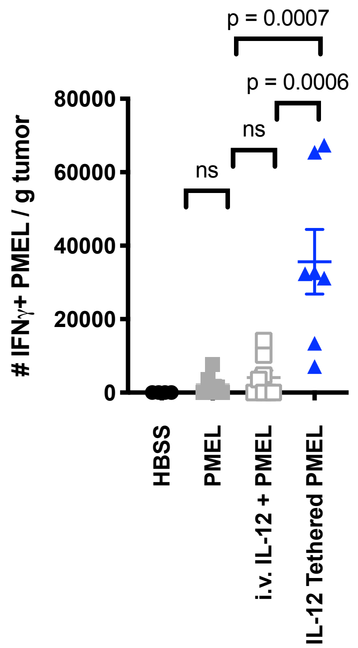

Figure S10. Number of IFNγ-expressing PMEL T cells in TME. Enumeration of IFNγ-expressing PMEL T cells in B16-F10 tumors 4 days after treatment with 5×10^6 PMEL T cells alone, tethered with CD45-IL-12 immunocytokine (approximately 210 ng IL-12), or in combination with i.v. co-injected IL-12 (500 ng).

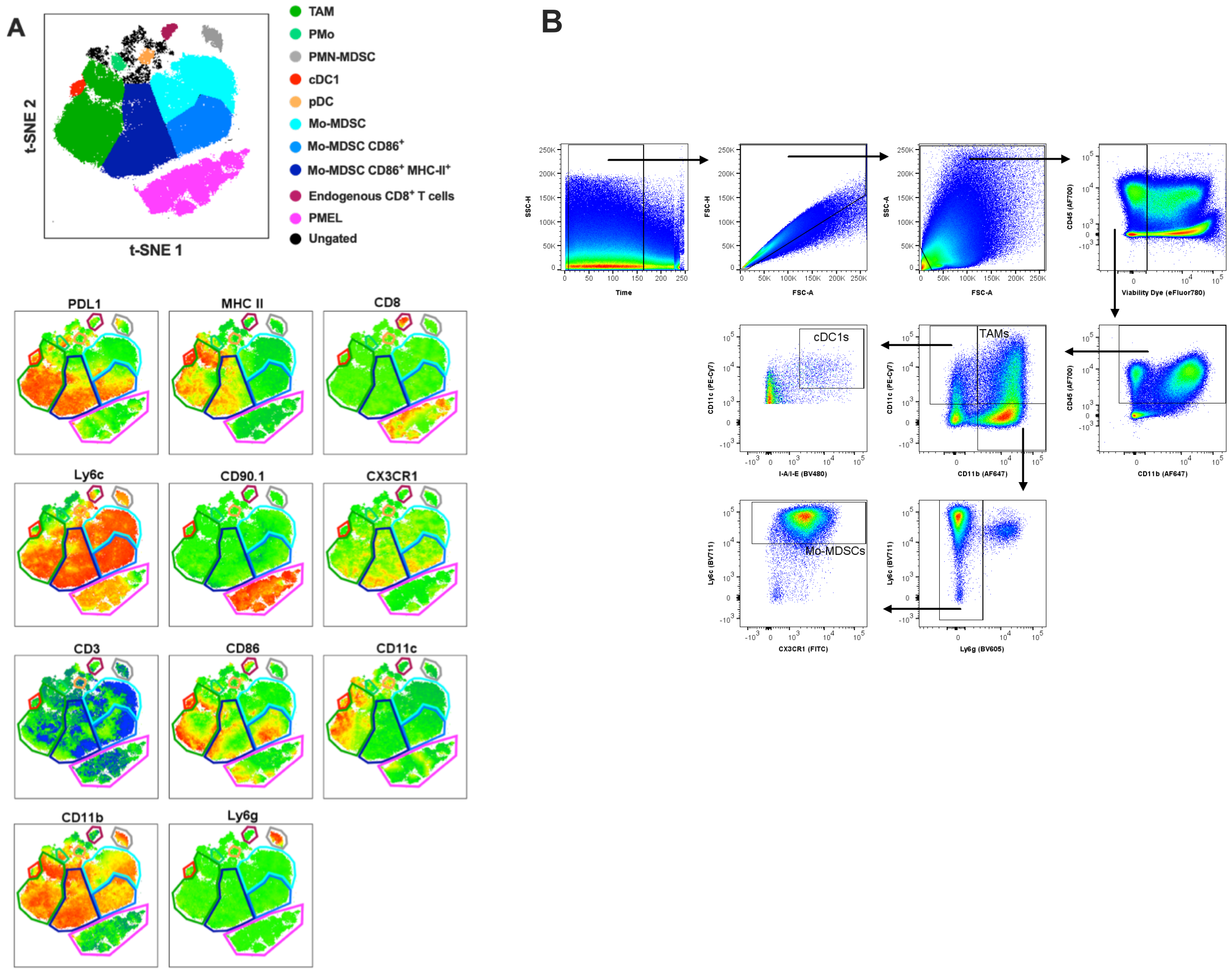

Figure S11. tSNE projections and gating strategy for tumor immune microenvironment study. (A) Relative clustering of various tumor immune cell types (top) and overlay of cell surface markers (bottom) in the tSNE space. **(B)** Gating strategy for tumor immune cell identification.

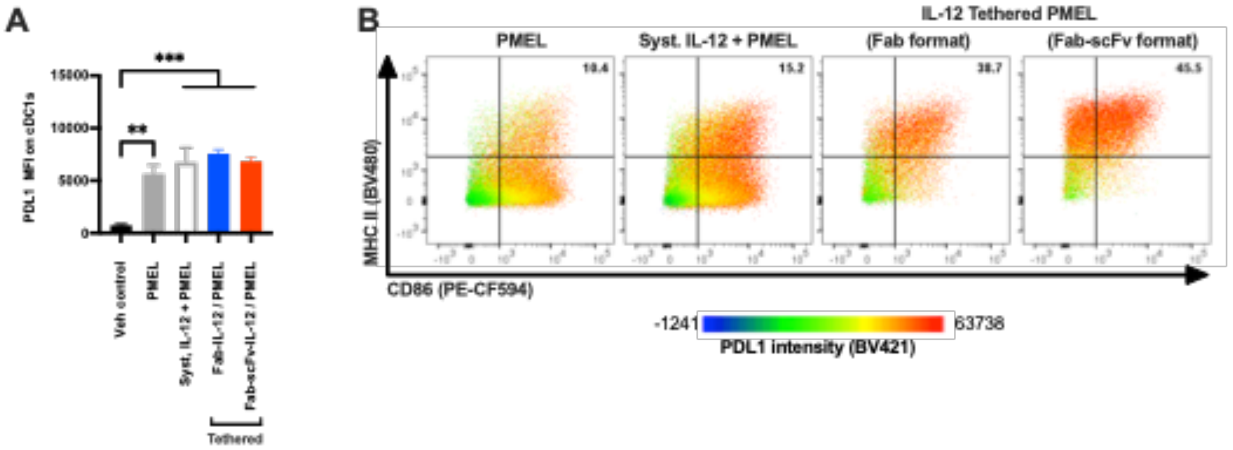

Figure S12. PDL1 expression on tumor immune cells. (A) PDL1 expression on cDC1 cells in the TME four days after treatment. **(B)** Representative plots for CD86 and MHC-II expression on Mo-MDSC with color based on PDL1 staining intensity.

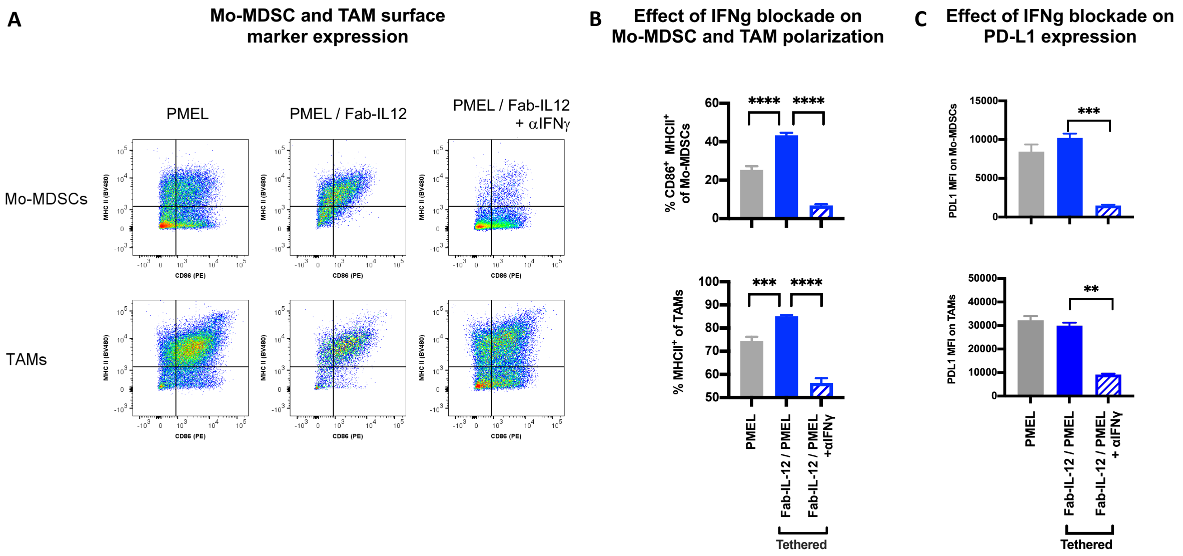

Figure S13. Effects of IFNγ neutralization on MHC-II, CD86, and PDL1 expression in Mo-MDSCs and TAMs. **(A)** Representative plots for MHC-II and CD86 expression on Mo-MDSC and TAMs after treatment. **(B)** Effect of IFNγ neutralization on Mo-MDSC activation (top) or M1-like MHC-II$^+$ TAMs (bottom). **(C)** Effect of IFNγ neutralization on PDL1 expression on Mo-MDSCs or TAMs. All treatment groups received 5×10^6 PMEL T cells alone or loaded with CD45-tethered IL-12, TME effects analyzed three days after adoptive transfer. For IFNγ neutralization, antibody clone XMG1.2 was dosed intraperitoneally at 300 μg on the same day as PMEL T cell adoptive transfer and repeated 2 days later.

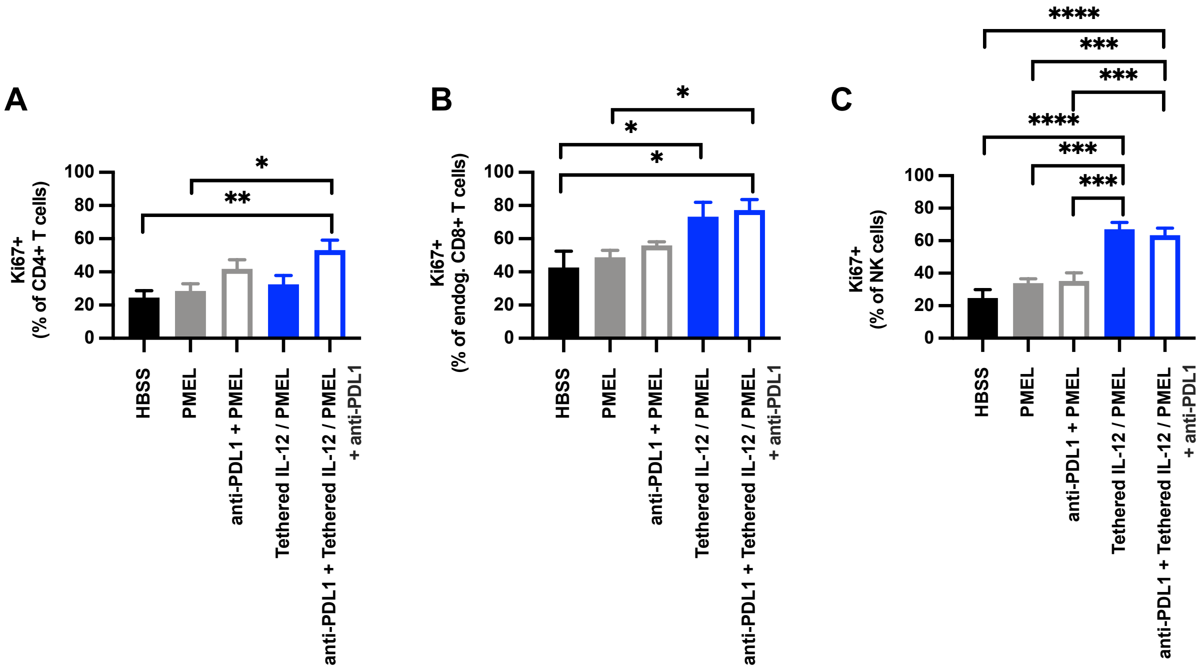

Figure S14. Proliferation of endogenous lymphocytes induced by IL-12 tethered T cells in combination with PDL1 blockade. (A-C) Percentages of Ki-67-positivity among **(A)** CD4 T cells, **(B)** endogenous CD8 T cells, and **(C)** NK cells in tumors four days after transfer of 5×10^6 PMEL T cells alone or tethered with IL-12 prior to ACT and with or without anti PD-L1 neutralizing antibody treatment. For PD-L1 neutralization, 200 µg antibody clone 10F.9G2 was dosed intraperitoneally 0 and 3 days after PMEL T cell adoptive transfer.

Supplementary Table 1. Antibodies for in vivo PMEL T cell profiling.

Species reactivity	Antigen	Fluorophore	Clone	Supplier
Mouse	CD45	BV510	30-F11	BioLegend
Mouse	CD4	AF488	GK1.5	BioLegend
Mouse	CD8a	BV605	53-6.7	BioLegend
Mouse	CD90.1	AF700	OX-7	BioLegend
Mouse	IFNγ	PE	XMG1.2	BioLegend
N/A	Zombie violet (viability dye)	BV421	N/A	BioLegend

Supplementary Table 2. Antibodies for tumor immune microenvironment profiling.

Species reactivity	Antigen	Fluorophore	Clone	Supplier
Mouse	PDL1 (CD274)	BV421	MIH5	BD Biosciences
Mouse	I-A/I-E (MHCII)	BV480	M5/114.15.2	BD Biosciences
Mouse	Ly6g	BV605	1A8	BioLegend
Mouse	CD8a	BV650	53-6.7	BD Biosciences
Mouse	Ly6c	BV711	HK1.4	BioLegend
Mouse	CD90.1	BV786	OX-7	BD Biosciences
Mouse	CX3CR1	FITC	SA011F11	BioLegend
Mouse	CD3e	BB700	145-2C11	BD Biosciences
Mouse	CD86	PE-dazzle594	GL-1	BioLegend
Mouse	CD11c	PE-Cy7	N418	BioLegend
Mouse	CD11b	AF647	M1/70	BD Biosciences
Mouse	CD45	AF700	30-F11	BD Biosciences
N/A	Viability Dye	eFluor780	N/A	Thermofisher Scientific
